# Chronic hyperglycemia and cardiovascular dysfunction: an in-depth exploration of metabolic and cellular pathways in type 2 diabetes mellitus

**DOI:** 10.1186/s40842-025-00247-3

**Published:** 2025-12-12

**Authors:** Araiz Hussain

**Affiliations:** Department of Pharmacy, Benazir Bhutto Shaheed University Lyari, Karachi, Pakistan

**Keywords:** Type 2 diabetes mellitus, Hyperglycemia, Cardiovascular disease, Polyol pathway, Advanced glycation end-products, Protein kinase C, Oxidative stress, Hexosamine biosynthetic pathway, Epigenetic modifications

## Abstract

Chronic hyperglycemia is the defining feature of type 2 diabetes mellitus (T2DM) and a central driver of its long-term complications, including microvascular and macrovascular diseases. Among these, cardiovascular disease (CVD) remains the leading cause of morbidity and mortality in individuals with T2DM. Persistent glucose elevation activates multiple interrelated biochemical pathways, including the polyol pathway, formation of advanced glycation end-products, protein kinase C activation, oxidative stress via several cellular mechanisms, as well as the hexosamine biosynthetic pathway. These processes collectively promote excessive oxidative damage, endothelial dysfunction, inflammation, and adverse cardiac remodeling, ultimately accelerating cardiovascular damage. Research has further revealed that hyperglycemia imprints long-lasting molecular changes through metabolic memory, largely mediated by epigenetic mechanisms such as DNA methylation, histone modifications, and non-coding RNAs, and sustaining vascular dysfunction even after glycemic control. Building on both classical and emerging evidence, this review synthesizes advances in understanding hyperglycemia-induced vascular damage and highlights promising therapeutic targets within these pathways. Strategies under investigation include inhibitors of enzymatic reactive oxygen species sources, modulators of mitochondrial dynamics and function, regulators of protein kinase C, blockers of glycation pathways, and epidrugs targeting epigenetic modifications. By integrating these mechanistic insights with therapeutic innovation, the field is shifting from a narrow focus on glucose lowering to a broader approach aimed at preventing and potentially reversing the vascular complications of diabetes. This perspective emphasizes the urgent need for pathway-specific therapeutic strategies to effectively address the cardiovascular burden of T2DM.

**Graphical Abstract:**

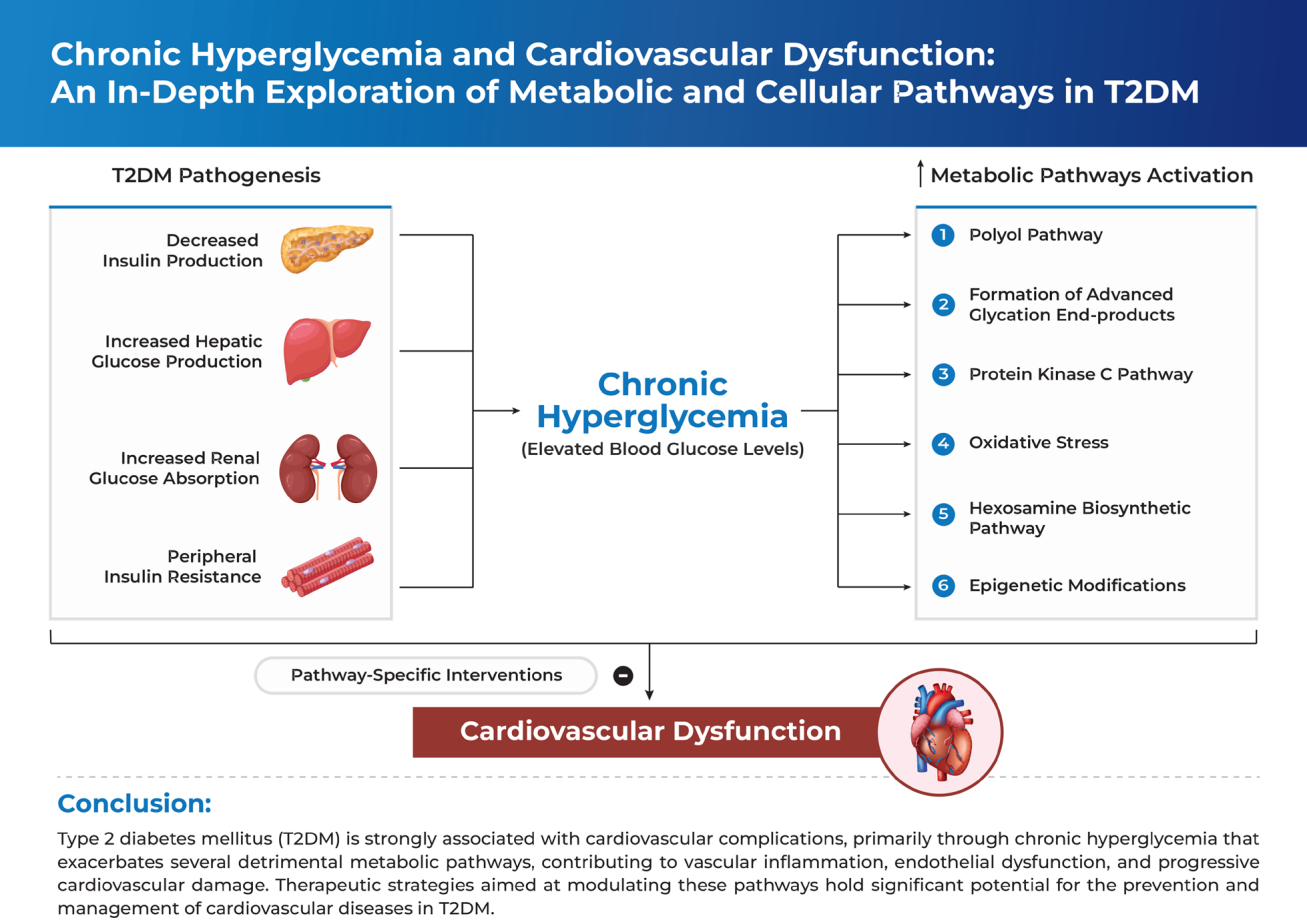

## Introduction

**Fig. 1:**
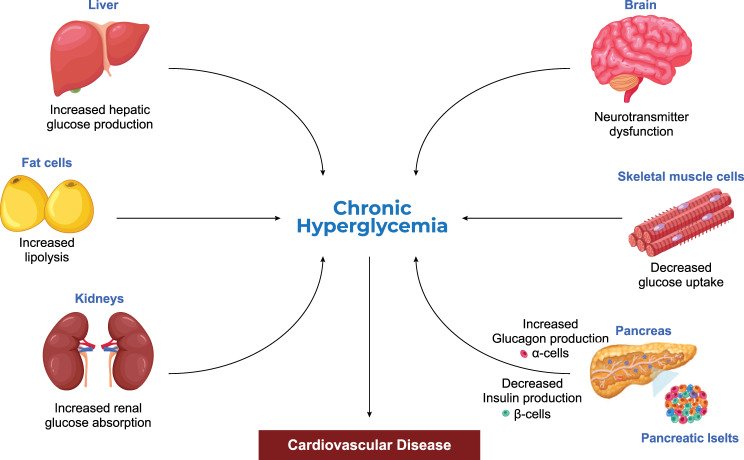
Pathogenesis and systemic mechanisms underlying the development of chronic hyperglycemia in T2DM. *Designed using elements from Freepik*

Diabetes mellitus (DM) is a complex metabolic disorder characterized by chronic hyperglycemia resulting from impaired insulin secretion, insulin resistance, or both [1]. Insulin is a naturally occurring endocrine hormone produced by pancreatic β-cells that regulates blood glucose by facilitating its uptake into the liver, muscles, and adipose tissue, and supporting metabolic processes [2]. If a prolonged insulin deficiency, insulin resistance, or both are not effectively controlled, they can lead to severe and potentially fatal health conditions, including cardiovascular diseases (CVD), diabetic nephropathy, diabetic neuropathy, and eye disorders, particularly those affecting the retina, leading to vision impairment and even blindness [3]. DM is primarily categorized into type 1 diabetes mellitus (T1DM) and type 2 diabetes mellitus (T2DM). T1DM is recognized as an autoimmune disorder resulting from the pancreatic β-cells destruction, eventually leading to impaired insulin secretion and hyperglycemia. Among all the cases of DM, only ~10% are diagnosed with T1DM. However, it is the predominant type of DM found in pediatrics and adolescent under 15 years of age, with over 500 million kids currently affected worldwide [4]. In contrast, T2DM is the most frequently observed type in adults aged 20–80, accounting for ~90% of the total DM cases [3–6]. It involves several complex mechanisms that contribute to hyperglycemia, the most significant of which include impaired insulin production by β-cells, increased production of glucagon through pancreatic α-cells, development of resistance of muscles and fat tissues to insulin activities, progression of cerebral neurotransmitter dysfunction, elevated hepatic glucose production, and increased lipolysis, as well as renal glucose reabsorption (Fig. 1) [7]. Once chronic hyperglycemia progresses to T2DM, a number of microvascular and macrovascular complications are concurrently established, with cardiovascular diseases (CVDs) being among the major contributors to morbidity and mortality in affected individuals [8].

### Epidemiology of DM and diabetes-related CVD mortality

**Fig. 2:**
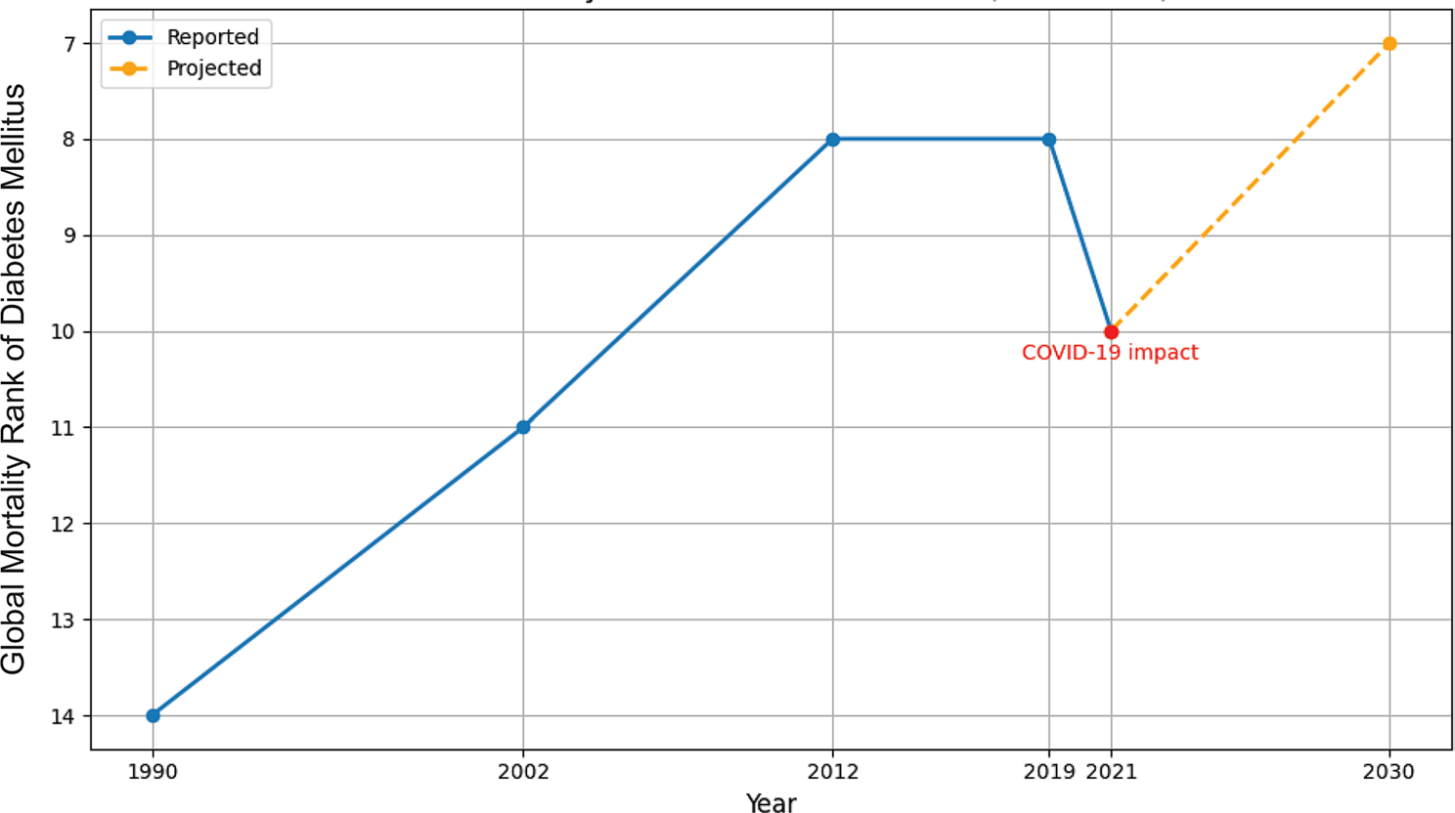
Global mortality rank of diabetes mellitus (1990–2030) based on global burden of disease data and WHO reports

Recognized as one of the most common metabolic disorders, DM represents a major global public health burden due to its associated complications and rising incidence [1, 6]. According to the International Diabetes Federation (IDF), the global prevalence of DM in adults aged 20–79 years has more than tripled since 2000, from approximately 151 million (4.6% of the global population at the time) to 537 million (10.5%) in 2021. Projections further estimate that the number will rise to 643 million (11.3%) by 2030 and 783 million (12.2%) by 2045, respectively [3]. DM has also demonstrated a significant upward trajectory in global mortality rankings over the past three decades (Fig. 2). The Global Burden of Disease (GBD) study documents DMs rise from the 14th leading cause of death in 1990 to 8th leading cause in 2019, with a temporary decline to 10th in 2021 attributable to mortality pattern disruptions during the COVID-19 pandemic [9]. Complementary data from the World Health Organization (WHO) demonstrate that DM rose from the 11th leading cause of death in 2002 to 8th leading cause in 2012, with current projections indicating its ascension to 7th position by 2030 [10, 11], demonstrating the growing burden of DM across both morbidity and mortality parameters.

**Fig. 3:**
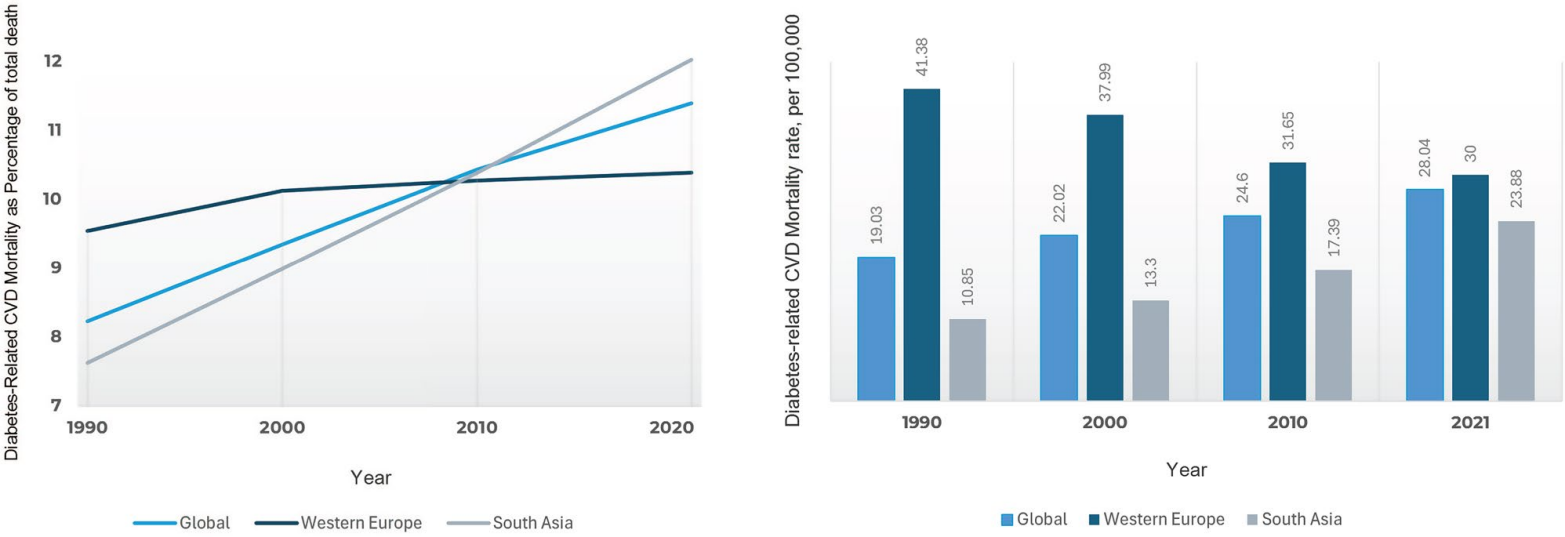
Diabetes related CVD mortality as percentage of total death and CVD mortality rate, per 100,000 according to the global burden disease 2021 study

CVD is the major macrovascular complication associated with T2DM, with two- to three-fold increased risk in T2DM individuals compared to non-diabetic population [12, 13], accounting for approximately 70% of global deaths among patients with T2DM [14, 15]. The most recent GBD 2021 study highlights a concerning global rise in CVD mortality associated with high fasting plasma glucose (HFPG), alongside marked regional diversity (Fig. 3) [16]. Between 1990 and 2021, the number of HFPG-related CVD deaths increased more than doubled globally (from 1.02 million to 2.21 million), with mortality rates rising from 19.03 to 28.04 per 100,000 population. HFPG accounted for a growing share of overall CVD mortality, increasing from 8.2% to 11.4% during this period. South Asia exhibited the steepest rise, with HFPG-attributable CVD deaths rising nearly fourfold (from 118,600 to 441,000) and mortality rates more than doubling from 10.8 to 23.9 per 100,000, while the proportion of CVD deaths due to HFPG increased from 7.6% to 12.0%. Conversely, Western Europe showed relative success in mitigating HFPG-related CVD mortality, achieving a 27.5% decline in mortality rates (from 41.4 to 30.0 per 100,000) and maintaining a stable contribution of HFPG to total CVD deaths at around 10% (9.5 to 10.4%), reflecting effective integration of glycemic control into broader cardiovascular prevention frameworks [16].

Although CVD in individuals with T2DM arises from a collection of interrelated risk factors, chronic hyperglycemia remains a fundamental and independent contributor to its pathogenesis. Persistent elevations in blood glucose initiate a series of metabolic and cellular disruptions that compromise vascular integrity as well as myocardial function. A comprehensive understanding of these hyperglycemia-driven mechanisms is therefore critical for elucidating the molecular basis of diabetic cardiovascular complications and for advancing targeted therapeutic strategies. To provide such understanding of this relationship, the author conducted a targeted literature search using PubMed and Google Scholar for relevant studies. The search emphasized original research and review articles addressing chronic hyperglycemia, cardiovascular complications, underlying pathophysiological mechanisms, and potential therapeutic targets of hyperglycemia driven CVDs in T2DM. The retrieved studies were critically examined and synthesized to provide an integrated and evidence-based understanding of the topic.

## Pathophysiological mechanisms of hyperglycemia-induced vascular damage

### The polyol pathway

**Fig. 4:**
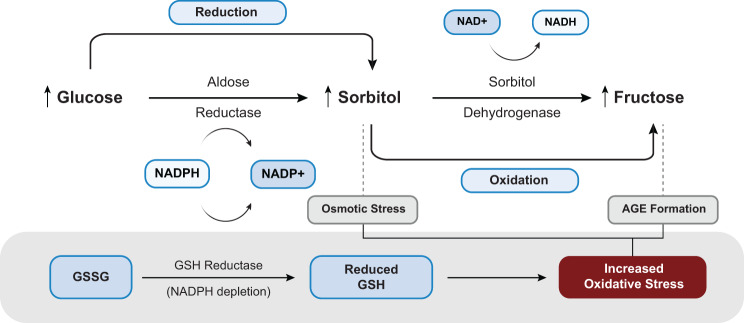
Increased glucose flux through the polyol pathway leads to redox imbalance and elevated oxidative stress. Abbreviations: NAD+, nicotinamide adenine dinucleotide; NADH, nicotinamide adenine dinucleotide (reduced); NADP+, nicotinamide adenine dinucleotide phosphate; NADPH, nicotinamide adenine dinucleotide phosphate (reduced); GSSG, oxidized glutathione; GSH, reduced glutathione; AGE: advanced glycation end-products

Several studies have linked elevated intracellular glucose levels to increased sorbitol accumulation, mediated by the sequential actions of aldose reductase (AR) and sorbitol dehydrogenase (SDH) through the polyol pathway. This pathway is accountable for the enzymatic transformation of intracellular glucose to sorbitol with the help of AR, after which the resulting sorbitol is further oxidized to fructose by SDH (Fig. 4). The initial step of this pathway requires NADPH, and thus, increased activation of this step leads to reduced availability of intracellular NADPH [17].

#### NADPH depletion and impaired antioxidant defense

It is estimated that around 3% of glucose is metabolized through the polyol pathway under normal physiological conditions. However, in hyperglycemia, this percentage can rise to over 30%, leading to a significant NADPH deficiency [18]. Among the major outcomes of NADPH shortage, increased risk of free radicals-induced oxidative stress due to reduced production of key antioxidants like Glutathione (GSH), holds a great significance. The synthesis of GSH is facilitated by the enzyme GSH reductase, which requires NADPH as a cofactor for its activity [17]. Studies have demonstrated that increased polyol pathway activity enhances NADPH consumption at higher rates, wherein AR and the antioxidant enzyme GSH reductase utilize the same cytoplasmic NADPH pool in competition [19]. In case of NADPH insufficiency, the activity of GSH reductase decreases, leading to reduced intracellular GSH levels and elevated levels of reactive oxygen species (ROS) under hyperglycemic conditions. This disrupts the cellular redox balance, ultimately increasing the chance of developing oxidative stress and associated cellular damage [17]. NADPH is also an essential factor for the production of nitric oxide (NO) from arginine, with the help of NO synthase as a catalytic agent. Its depletion disrupts and reduces NO production, thereby increasing the risk of endothelial dysfunction and contributing to cardiovascular complications [20]. Increased glucose flux through the pathway further disrupts normal cellular metabolism, promoting oxidative stress, advanced glycation end-products (AGEs) formation, DNA damage, and ultimately, apoptosis [21].

#### AR-derived metabolites in CVD pathogenesis

The principal enzyme of the polyol pathway, AR, has been identified as a critical mediator of oxidative and inflammatory signaling, as well as endothelial dysfunction, triggered by growth factors, cytokines, and hyperglycemia [21], preventable through AR inhibition [19]. The resulting intracellular accumulation of sorbitol induces osmotic stress, while its downstream metabolite, fructose, undergoes glycation reactions that lead to the formation of AGEs, further exacerbating oxidative damage and inflammatory processes through a series of reactions [22, 23]. Daniel et al. [24] in 2021, following clinical evaluation of the association between myocardial fructose and sorbitol levels and indicators of cardiac dysfunction in diabetic patients, reported that myocardial sorbitol and fructose levels were significantly elevated in patients with T2DM. Experimental findings further revealed that increased expression of fructose-metabolizing enzymes and fructose exposure in cardiomyocytes promoted glycolytic activity and lipid accumulation, strongly suggesting a direct role of the polyol pathway in diabetic cardiac dysfunction [24]. It has also been demonstrated that elevated cardiac fructose levels, resulting from both dietary intake, as well as increased polyol pathway activation, are associated with oxidative stress and metabolic dysfunction in the heart. This fructose accumulation was shown to promote lipid deposition, inflammation, and contractile dysfunction in cardiomyocyte [25]. Similarly, Heianza et al. [26] reported that elevated plasma sorbitol levels are associated with a higher long-term risk of coronary heart disease (CHD) in women, independent of traditional cardiovascular risk factors. These finding further support the concept that hyperglycemia-driven increased activation of the polyol pathway, with increased sorbitol and fructose production, contributes to an elevated cardiovascular risk in the diabetic population.

#### Disruption of NAD+/NADH redox equilibrium

Disruption of the NADH/NAD^+^ redox equilibrium has also emerged as one of the findings resulting from overactivation of polyol pathway, particularly during the second step catalyzed by SDH, where NAD^+^ is reduced to NADH through an electron transfer. Studies reveal that increased polyol pathway flux disrupts glycolysis in diabetic hearts, primarily due to competition between SDH and glyceraldehyde-3-phosphate dehydrogenase (GAPDH) for the cytosolic NAD^+^ pool [19]. Under hyperglycemic conditions, the polyol pathway serves as a principal contributor to elevated NAD^+^ consumption and excessive NADH generation, thereby disrupting the cytosolic free NADH/NAD^+^ ratio and subsequently damaging neural and vascular function [19, 27]. Elevated NADH levels intensify electron flux through the mitochondrial electron transport chain (ETC), resulting in electron overload and leakage, primarily at Complexes I and III, where premature interactions with molecular oxygen give rise to superoxide, hydrogen peroxide, and hydroxyl radicals [28]. Briefly, increased glucose flux through the polyol pathway depletes NADPH, leads to intracellular sorbitol accumulation, promotes fructose-mediated AGE formation, disrupts NADH/NAD^+^ ratio, and increases NADH generation, subsequently causing electron overload in the mitochondrial ETC and thereby collectively inducing oxidative stress that contributes to the development and progression of cardiovascular dysfunction in T2DM.

### Non-enzymatic glycation and AGE formation

**Fig. 5:**
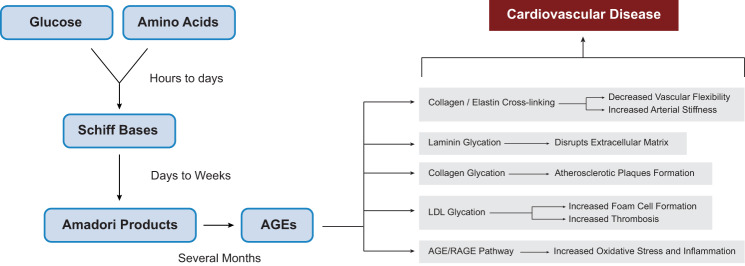
Formation of advanced glycation end-products and their vascular consequences in hyperglycemia. Abbreviations: AGEs, advanced glycation end-products; LDL, low-density lipoprotein; RAGE, receptors for advanced glycation end-products

Non-enzymatic glycation is a spontaneous condensation reaction in which free-reducing sugars, including glucose, interact with the N-terminal or free amino groups of proteins, lipids, and nucleic acids, through nucleophilic addition reactions, resulting in the formation of unstable Schiff bases. These bases then undergo additional rearrangements to produce more stable Amadori products. While most of the Amadori products remain relatively stable, a small proportion undergoes further irreversible rearrangements and chemical modifications, including dehydration and oxidation, ultimately generating a heterogeneous group of protein-bound molecules called AGEs (Fig. 5) [29–31]. The conversion of Amadori products to AGEs is a slow process and occurs gradually over several months [32].

#### AGEs cross-linking with proteins

Once AGEs are formed, they can cause multiple detrimental effects, leading to alteration of cardiovascular structure and inflammation of blood vessels, ultimately affecting the health of the cardiovascular system [32]. AGEs cross-link with several extracellular proteins including collagen, laminin, and elastin, modify them by glycation, and directly contributing in the initiation of atherosclerosis. The AGE-induced modification of extracellular proteins alters the structural, as well as the functional properties of healthy tissues and promotes inflammatory responses. Collagen, laminin, and elastin are fundamental structural proteins present in the basement membranes and connective tissues. Because of their extended half-life and a particular composition of amino acids, these proteins are highly vulnerable to glycation by AGEs [33]. AGEs cross-linking with extracellular proteins such as collagen and elastin results in significantly reduced vascular flexibility and increased stiffness of blood vessels [34]. Schram et al. [35] in the Hoorn study, reported a substantial increase in central arterial stiffness among individuals with T2DM. Glycated collagen disrupts endothelial cell function and facilitates the accumulation of atherosclerotic plaques, while the glycation of laminin by AGEs reduces its binding affinity to type IV collagen and impairs the formation of matrix-like structures. As a result, the structural integrity and functionality of the extracellular matrix in arteries disrupt, which leads to impaired vascular health and an elevated risk of cardiovascular complications [33, 36, 37].

#### AGEs and foam cell formation

AGEs also target lipoproteins, such as low-density lipoproteins (LDL), promoting its glycation through oxidative modifications. In T2DM, LDL is frequently found in its glycated form [36]. When LDL undergoes glycation, its structure is altered, which impairs its recognition and uptake by LDL receptors, leading to higher concentrations in the bloodstream. Consequently, the uptake of glycated LDL by monocytes and macrophages is decreased, which contributes substantially to foam cell formation [38]. Recently generated foam cells begin to build up within the arterial intima, triggering the accumulation of atherosclerotic lesions during the initial phases [39]. Furthermore, glycated LDLs have also been found to reduce the production of tissue plasminogen activators within endothelial cells, thereby elevating the risk of thrombosis [33].

#### AGEs serum levels and diabetic CVD

Patients with DM typically exhibit 20%-30% higher circulating levels of AGEs compared to healthy controls, while those diabetic CVDs can show increases of up to 40%-100% [40]. The accumulation of AGEs has been associated with several cardiovascular complications, including atrial fibrillation (AF) [41], congestive heart failure (CHF) [42], and coronary artery disease (CAD) [43] in individuals with T2DM. Increased AGE levels may serve as significant biomarkers and predictors of heart failure and cardiovascular mortality in patients with T2DM, as their accumulation has been demonstrated in both atherosclerotic plaques and myocardial tissues [40]. Raposeiras-Roubin et al. [44] investigated the relationship between AGEs and AF by measuring plasma AGE levels and serum concentration of soluble receptors for AGEs (sRAGE) in 97 patients, including both groups of diabetic and non-diabetic patients. The results revealed higher concentrations of AGEs and sRAGE in AF patients, which correlated with atrial enlargement. Multivariate analysis identified both as independent predictors of AF, emphasizing their role in atrial arrhythmogenesis. Similarly, in a recent retrospective cohort study involving 362 patients with T2DM, increased levels of circulating AGEs and sRAGE isoforms were noted, with higher AGEs/cRAGE ratios linked to elevated all-cause mortality. Additionally, sRAGE was correlated with the occurrence of major adverse cardiovascular events (MACE) in those without prior events [45]. Whereas Kajikawa et al. [46] report that sRAGE functions as a counter-regulatory factor, activated to mitigate the vasotoxic effects of the AGE–RAGE axis. Several other studies have also highlighted the involvement of AGE accumulation in the development and progression of pre-existing CVD. For instance, Kilhovd et al. [40], after an 18-year follow-up study, demonstrated that increased AGE concentrations were significantly linked with CAD progression and total mortality, especially in females, despite adjusting for traditional risk factors. Pentosidine and Nε-(carboxymethyl)lysine (CML), the predominant serum AGEs, were reported by Kerkeni et al. [41] and Semba et al. [42] respectively, as independently associated glycated products correlated with higher rates of cardiovascular challenges and mortalities in diabetic patients, strongly supporting the concept that AGE accumulation worsens pre-existing cardiovascular conditions, accelerates vascular dysfunction, and promotes the progression of CVD.

#### The AGE-RAGE signaling pathway

The formation of AGEs also triggers activation of the receptor for AGEs (RAGE), which in turn exacerbates oxidative stress and inflammation through the stimulation of signaling pathways, such as NFκB and NADPH oxidase, thereby sustaining a self-perpetuating cycle of vascular and tissue injury [24]. The AGE-RAGE interaction activates several other signaling molecules including p21RAS, cdc42/rac, extracellular signal-regulated kinase (ERK)1/2, mitogen-activated protein kinase (MAPK), and Janus kinase (JAK)/STAT. These activations modulate cellular functions and triggers several proinflammatory and procoagulant gene pathways, which ultimately contribute to adverse cardiovascular outcomes [38]. Moreover, the interaction of AGEs with RAGE on endothelial cells leads to increased production of asymmetric dimethylarginine (ADMA), an endogenous inhibitor of endothelial nitric oxide synthase (eNOS) and a potent biomarker of cardiovascular disease progression [47]. Lu et al. [48] analyzed 1,320 patients undergoing coronary angiography and categorized them into four groups on the basis of T2DM presence and significant CAD (≥70% stenosis). The findings revealed that diabetic patients of CAD had significantly increased levels of glycated albumin, inflammatory markers (hsCRP, TNF, IL-6), and decreased levels of endogenous secretory RAGE (esRAGE) in comparison with other groups. Glycated albumin and esRAGE were independently associated with CAD severity and the number of diseased arteries, highlighting their role in CAD progression.

### Protein kinase C pathway

**Fig. 6:**
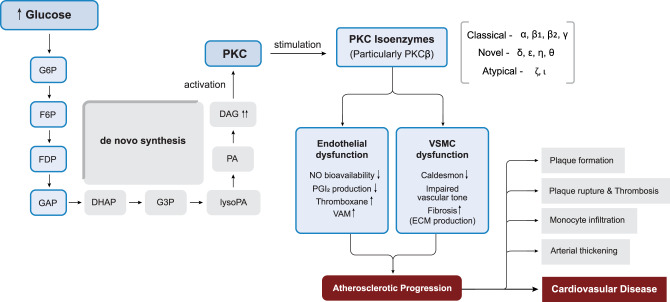
Hyperglycemia-induced PKC activation and atherosclerotic progression. Abbreviations: G6P, glucose-6-phosphate; F6P, fructose-6-phosphate; FDP, fructose-1,6-bisphosphate; GAP, glyceraldehyde-3-phosphate; DHAP, dihydroxyacetone phosphate; G3P, glycerol-3-phosphate; lysoPA, lysophosphatidic acid; PKC, protein kinase C; DAG, diacylglycerol; PA, phosphatidic acid; NO, nitric oxide; PGI₂, prostacyclin; VAM, vascular adhesion molecules; VSMC, vascular smooth muscle cell; ECM, extracellular matrix

Hyperglycemia-induced activation of the protein kinase C (PKC) pathway represents a critical mechanism in T2DM that associates metabolic disturbances to impaired vascular signaling and cardiovascular complications. Elevated blood glucose levels influence the function of signal transduction pathways, particularly through the initiation of diacylglycerol (DAG) and PKC [44]. Under diabetic conditions, DAG accumulation occurs through multiple pathways. One of the primary mechanisms involves enhanced *de novo* synthesis, where the glycolytic intermediate dihydroxyacetone phosphate (DHAP) is reduced to glycerol-3-phosphate (G3P) and subsequently undergoes stepwise acylation. This process is further amplified by the inhibition of GAPDH, an enzyme responsible for converting glyceraldehyde-3-phosphate (GAP) within the glycolytic energy production pathway. Consequently, the accumulation of GAP increases the flux of DHAP into DAG biosynthesis, thereby enhancing the PKC activation (Fig. 6) [49]. PKC is considered as a family of isozymes divided into classical, novel, and atypical PKCs [49]. The stimulation of one or multiple of these PKC isoforms results in various biological processes, including modifications in cell proliferation and differentiation, ion transport across membranes, glucose and lipid metabolism, smooth muscle contraction, and regulation of gene expression [50]. These biological events can affect multiple physiological processes within the CVS, leading to both chronotropic and ionotropic effects.

#### PKC pathway in diabetic cardiovascular complications

The hyperglycemic activation of PKC may serve as an associated mechanism through which oxidative stress and AGEs exert their detrimental effects on CVS [44], with its activation further stimulated by oxidative stress, angiotensin II, platelet-derived growth factor (PDGF), and vascular endothelial growth factor (VEGF), collectively intensifying its pathological impact. This activation initiates a cascade of detrimental downstream effects that impair vascular function and compromise tissue integrity [22]. Many studies have connected the activation of PKC pathway to several cardiovascular conditions, with considerable attention directed towards cardiac ischemia [47]. Geraldes and King [48] conducted a comprehensive review highlighting that elevated blood glucose levels and DM lead to vascular DAG accumulation, which activates PKC signaling pathways. This activation plays a significant role in the development of diabetic macrovascular complications, including cardiomyopathy and accelerated atherosclerosis. The study emphasizes that specific PKC isozymes, triggered by lipid metabolites and hyperglycemia, contribute to vascular abnormalities such as fibrosis, dysfunction of endothelial and vascular smooth muscle cells (VSMC), monocyte activation, cytokine imbalance, excessive extracellular ROS production, and impaired vascular insulin signaling that contribute significantly to DM associated vascular dysfunction [48]. PKC activation can also modulate muscle tone and blood vessel contraction by reducing caldesmon levels, a protein that inhibits smooth muscle contraction, resulting in increased muscle contraction and decreased blood flow, ultimately impairing normal tissue perfusion [51].

#### PKC-β as a mediator of macrovascular dysfunction

Among PKC isoforms, PKC-β is predominantly activated in diabetic hyperglycemia and is a key mediator of associated macrovascular dysfunction. The activation of PKC-β impairs endothelium-dependent vasodilation by reducing NO bioavailability [52] and contributing to increased ROS production [53]. In addition, it promotes endothelial proliferation by activating phospholipase A2 and stabilizing vascular endothelial growth factor (VEGF) mRNA expression, thereby further contributing to vascular remodeling and dysfunction. Evidence from both experimental models and clinical studies indicates that selective inhibition of PKC-β with Ruboxistaurin (RBX) effectively restores vascular reactivity and mitigates hyperglycemia-induced endothelial dysfunction [52, 53]. Hyperglycemic stimulation of PKC-β in atrial cardiomyocytes activates the NF-κB/TGF-β signaling pathway, leading to increased expression of pro-inflammatory and pro-fibrotic mediators such as TNF-α, TGF-β, and signaling molecules like p-IκB, p38 MAPK, Cav1.2, and NCX. This signaling cascade drives atrial structural and electrical remodeling, including fibrosis and altered calcium handling, which increases susceptibility to AF. Pharmacological inhibition of PKC-β with RBX, as well as genetic suppression via small interfering RNA, attenuated these effects and led to a decreased incidence of AF in diabetic experimental models [54].

#### PKC-driven vascular inflammation and atherosclerotic progression

PKC activation promotes a pro-inflammatory, atherogenic environment in diabetic vasculature by enhancing cytokine activity and endothelial adhesion molecule expression [55]. Elevated circulating IL-18 levels have been associated with accelerated atherosclerosis and the stimulation of pro-inflammatory cytokines that contribute to plaque destabilization in DM. Additionally, IL-18 enhances monocyte adhesion to endothelial cells and upregulates VCAM-1 expression, further promoting vascular inflammation and instability [55]. Inhibition with RBX effectively mitigated these alterations in streptozotocin-induced (STZ-induced) diabetic mice by reducing cholesteryl ester accumulation, limiting macrophage infiltration, and restoring IL-18 binding proteins expression, thereby attenuating the progression of atherosclerotic plaques [56]. Research involving human endothelial cells, *U*-931 cells (human histiocytic lymphoma), and hepatic G2 cells has also highlighted particular effects of PKC isozymes at various phases of atherosclerosis advancement [47]. Harja et al. [57] demonstrated that inhibition of PKC-β in ApoE-deficient mice attenuated atherosclerotic plaque formation by suppressing the expression of the transcription factor Egr-1. Similarly, in diabetic ApoE-deficient mice, PKCβ has been demonstrated to contribute to the acceleration of atherosclerosis by modulating the IL-18/IL-18BP axis and MAP kinase signaling pathways, thereby promoting VCAM-1 expression, enhancing macrophage adhesion, impairing endothelial function, and amplifying inflammatory responses in both the aorta and macrophages [58, 59].

### Oxidative stress

**Fig. 7:**
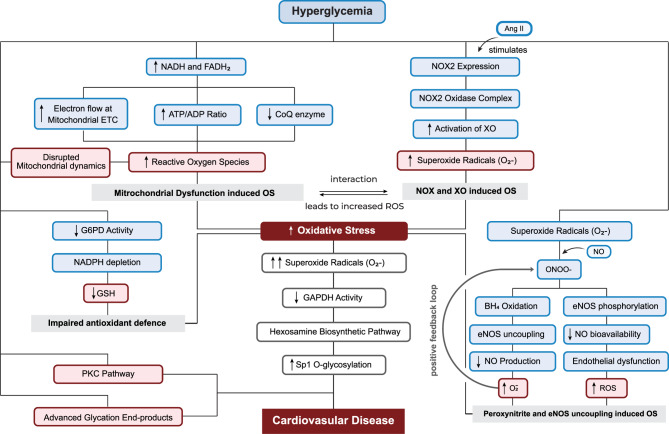
Mechanisms of oxidative stress in hyperglycemia-induced cardiovascular disease. Hyperglycemia leads to increased production of ROS through both mitochondrial dysfunction and NOX-dependent pathways. Elevated NADH/FADH₂ levels and disrupted ETC activity promote mitochondrial ROS generation, while angiotensin II stimulates NOX expression and XO activity. Oxidative stress is further amplified by NADPH depletion, reduced GSH levels, impaired antioxidant defense, and activation of secondary mechanisms including PKC signaling, AGE formation, and the HBP. Additionally, ONOO^−^ formation contributes to endothelial dysfunction through eNOS uncoupling, reduced NO bioavailability, and a positive feedback loop exacerbating ROS accumulation. Abbreviations: AGEs, advanced glycation end-products; Ang II, angiotensin II; ATP, adenosine triphosphate; BH₄, tetrahydrobiopterin; CoQ, coenzyme Q; eNOS, endothelial nitric oxide synthase; ETC, electron transport chain; FADH₂, flavin adenine dinucleotide (reduced form); G6PD, glucose-6-phosphate dehydrogenase; GAPDH, glyceraldehyde-3-phosphate dehydrogenase; GSH, glutathione; NADH, nicotinamide adenine dinucleotide (reduced form); NADPH, nicotinamide adenine dinucleotide phosphate (reduced form); NO, nitric oxide; NOX, NADPH oxidase; ONOO^−^, peroxynitrite; PKC, protein kinase C; ROS, reactive oxygen species; OS, oxidative stress; Sp1, specificity protein 1; XO, xanthine oxidase

A number of studies have identified a relationship between oxidative stress and the progression of cardiovascular complications induced by hyperglycemia [22, 60–64], which is strongly linked to decreased insulin sensitivity and insufficient insulin production, ultimately contributing to the development of T2DM itself [62]. Oxidative stress is generally considered as an imbalance between the production of free radicals, including ROS, and the body’s ability to neutralize them through various antioxidant defenses. Elevated levels of free radicals may contribute to vasoconstriction, intensified coagulation, and increased levels of adhesion molecules [65]. ROS are primarily generated by the oxidation of glucose, and thus, hyperglycemia further elevates the risk of tissue damage due to oxidative stress [56]. Multiple mechanisms have been implicated in the elevation of oxidative stress under hyperglycemic conditions. These include alterations in mitochondrial dynamics, electron overload within the mitochondrial ETC, activation of NADPH oxidase and xanthine oxidase, formation of peroxynitrites, and eNOS uncoupling (Fig. 7).

#### Disruption of mitochondrial dynamics

Mitochondria are double-membrane organelles responsible for generating ATP through the utilization of glucose and oxygen. To maintain mitochondrial dynamics, they continuously undergo fusion and fission reactions, which are essential for recycling and biogenesis, sustaining energy production, and regulating the ROS balance [66]. Mitochondrial fusion is primarily regulated by the proteins mitofusin-1 (Mfn1), mitofusin-2 (Mfn2), and optic atrophy protein 1 (Opa1), whereas mitochondrial fission is largely mediated by dynamin-related protein 1 (Drp1), mitochondrial fission 1 protein (Fis1), and mitochondrial fission factor (Mff) [67]. Mitochondrial fission facilitates the separation and isolation of damaged mitochondrial segments for subsequent removal via mitophagy, whereas fusion serves as a compensatory process that restores mitochondrial integrity by reintegrating fragmented organelles into the mitochondrial network [66]. Increasing evidence indicates that hyperglycemia and insulin resistance in T2DM disrupt mitochondrial dynamics by enhancing fission processes while suppressing fusion, thereby contributing to mitochondrial dysfunction. For instance, hyperglycemia in diabetic hearts increases Drp1 expression, which drives mitochondrial fission, elevates oxidative stress, and impairs both mitochondrial and cardiac function through suppression of SIRT1/PGC-1α signaling. Pharmacological treatment with melatonin counteracted these effects by enhancing SIRT1 and PGC-1α activity, reducing Drp1 levels, lowering ROS production, and restoring mitochondrial and overall cardiac function [68]. Similarly, diabetic mouse hearts exhibited reduced expression of Mfn2 alongside increased mitochondrial fission, which was associated with impaired mitochondrial function and diabetic cardiomyopathy [69]. Furthermore, in diabetic mice, Piezo1 overactivation was associated with excessive mitochondrial fission and reduced fusion, causing disrupted mitochondrial dynamics, impaired cardiac function and increased fibrosis [70]. In parallel, human atrial tissues from patients with persistent AF and DM exhibited significantly reduced Mfn2 expression and disrupted sarcoplasmic reticulum-mitochondrial coupling, which were associated with mitochondrial dysfunction and contractile impairment [71].

Mitochondrial dynamics are further aggravated by increased ROS, which enhances the expression of fission-related proteins and induces post-translational modifications that drive mitochondrial fragmentation and apoptosis [72]. Mitochondrial ROS promote dysfunction by enhancing ubiquitination of AKAP121, which reduces DRP1 phosphorylation at Ser637 and alters OPA1 proteolytic processing, thereby driving excessive mitochondrial fission in a transgenic mouse model of cardiac lipotoxicity [73]. Mitochondrial ROS scavenging effectively restored mitochondrial morphology both in vivo and in vitro. Hyperglycemia has also been demonstrated to disrupt mitochondrial dynamics by promoting Drp1 phosphorylation at Ser616 and reducing its phosphorylation at Ser637, thereby shifting the balance toward excessive mitochondrial fission and consequent mitochondrial dysfunction, which contributes to the progression of cardiomyocyte hypertrophy in diabetic rats [74]. In the diabetic heart, excessive production of ROS disrupts calcium homeostasis, induces opening of the mitochondrial permeability transition pore (mPTP), and activates apoptotic pathways, processes that are central to the pathogenesis of diabetic cardiomyopathy and heart failure [75]. Likewise, in both diabetic patients and STZ-induced T2DM mice, hyperglycemia was associated with reduced expression of USP28 (a deubiquitinating enzyme), which led to disrupted mitochondrial dynamics, lipid accumulation, and progressive cardiac dysfunction, while cardiac-specific overexpression of USP28 ameliorated these effects. Mechanistically, USP28 directly interacts with PPARα, deubiquitinates and stabilizes it at Lys152, promoting transcription of Mfn2 to restore mitochondrial fusion, which mitigates ROS production, reduces fibrosis, and improves both systolic and diastolic function in models of diabetic cardiomyopathy [76]. Collectively, the findings indicates that enhanced mitochondrial fission coupled with diminished fusion represents a defining feature of CVDs and likely plays a central role in the pathogenesis of several cardiovascular complications including heart failure, diabetic cardiomyopathy, and atherosclerosis.

#### Mitochondrial electron transport chain

The mitochondrial ETC, comprising complexes I–IV within the inner mitochondrial membrane, facilitates the transfer of electrons from reduced substrates such as NADH, FADH₂, and cytochrome c to molecular oxygen, thereby sustaining oxidative phosphorylation [67]. In parallel, mitochondria represent the predominant source of intracellular ROS, with complexes I and III serving as principal sites of superoxide generation [77]. Under physiological conditions, a small proportion of electrons (approximately 2%) prematurely leak from the ETC prior to reaching complex IV, leading to the partial reduction of oxygen and the formation of superoxide [78]. In hyperglycemic states, excessive availability of electron donors increases NADH and FADH₂ levels, driving increased electron flux through the ETC and causing electron overload at Complexes I and III. This accelerates ATP synthesis and induces hyperpolarization of the mitochondrial membrane potential, which impairs electron transfer at Complex III. As a result, electrons accumulate at coenzyme Q, promoting incomplete oxygen reduction and excessive superoxide generation, thereby intensifying mitochondrial oxidative stress [28, 79]. The depletion of coenzyme Q, as well as the generation of superoxides, are considered as critical factors in mitochondrial dysfunction, which plays a significant role in the development of diabetic metabolic disorders [80]. Several studies have highlighted the significance of ROS in cardiomyocytes, predominantly generated by the ETC in mitochondria. Notably, Ide et al. [81] provided strong evidence that link mitochondrial ROS to heart failure myocytes, indicating their contribution to both structural damage and contractile dysfunction within the myocardium. Hyperglycemia-induced mitochondrial superoxide overproduction further enhances the activation of the hexosamine and PKC pathways and promotes the formation of AGEs. These processes collectively drive endothelial dysfunction, lipid peroxidation, and atherosclerosis, significantly contributing to the progression of diabetic CVD [80]. Additionally, increased production of superoxides suppresses the activity of glucose-6-phosphate dehydrogenase (G6PD), a key enzyme that regulates the pentose phosphate pathway (PPP) responsible for generating NADPH. The suppression of G6PD ultimately leads to deficiency of NADPH, which is responsible for the production of GSH as a cofactor, further disrupting the antioxidant defense system [80].

#### NADPH oxidase

Intracellular oxidative stress has been determined to emerge from several mechanisms, including elevated ROS [82]. Hyperglycemia leads to excessive ROS generation through overactivation of NADPH oxidase (NOX). The resulting ROS buildup disrupts the nuclear factor erythroid 2-related factor 2 (NRF2) signaling pathway, suppressing the expression of antioxidant and anti-inflammatory genes, and thereby intensifying oxidative stress [22]. NOX is the major source of ROS in cells such as VSMCs, endothelial cells, and fibroblasts [82]. Among the NOX isoforms, NOX1 to NOX5 are expressed in endothelial cells, with NOX2 being the primary source of pathological ROS linked to endothelial damage and atherosclerosis [63]. However, the involvement of NOXs in diabetic complications seems to be more intricate than merely enhancing ROS generation. In the context of macrovascular disease, a leading hypothesis suggests that the elevated cardiovascular risk associated with DM arises from hyperglycemia-driven acceleration of atherogenesis through increased ROS production. Supporting this, evidence from preclinical models indicates that NOX1-derived superoxide contributes to the progression of diabetic vasculopathy [83]. Angiotensin II (Ang II) strongly stimulates the production of ROS and contributes to vascular dysfunction. Research indicates that Ang II enhances NOX2 expression and facilitates the rapid assembly of its oxidase complex within endothelial cells [63]. Inhibiting NOX has been observed to prevent superoxide production induced by Ang II through xanthine oxidase (XO), another key source of ROS within cells, highlighting that Ang II activation of XO depends on NOX activity [84].

#### Xanthine oxidase

XO is a key enzyme involved in purine metabolism, facilitating the sequential oxidation of hypoxanthine to xanthine, and subsequently to uric acid. It transfers electrons directly to molecular oxygen (O₂), resulting in the generation of ROS, specifically superoxide anion (O₂∙^−^) through a one-electron reduction, and hydrogen peroxide (H₂O₂) via a two-electron reduction. Under physiological conditions, H₂O₂ is considered the predominant ROS produced during XO-mediated oxygen reduction [85]. XOR, which includes both XO and xanthine dehydrogenase (XDH), is considered to play a central role in the production of ROS and induction of vascular endothelial dysfunction in patients with T2DM [86]. In a clinical study conducted by Okuyama et al. [86], plasma XOR activity levels were found to be elevated in Japanese patients with T2DM compared to the general population. Serum XO levels have also been reported to be significantly elevated in diabetic patients [87], as well as found to be associated with specific cardiovascular complications, such as coronary artery spasm (CAS) [88].

#### Formation of peroxynitrite, and eNOS uncoupling

Oxidants and ROS are not only directed linked to CVD but may also play a crucial role in driving certain indirect aspects of vascular and cardiac dysfunction. For instance, under both experimental and pathological conditions, vascular impairment and reduced heart function are influenced by peroxynitrite (ONOO^−^), a potential oxidant generated through the chemical reaction between superoxide radicals (O₂^−^) and NO, capable of producing irreversible cytotoxic effects [63, 89]. ONOO^−^ also oxidizes the eNOS cofactor BH4, leading to eNOS uncoupling, which reduces NO production and increases O₂^−^ generation. This vicious cycle is further exacerbated by eNOS inactivation through phosphorylation, connecting ROS sources to impaired endothelial function and excessive ROS production [64]. eNOS inhibition is further mediated by the PKC pathway under hyperglycemic conditions, leading to reduced NO bioavailability, excessive ROS generation, and impaired vascular function [22].

### Hexosamine biosynthetic pathway

**Fig. 8:**
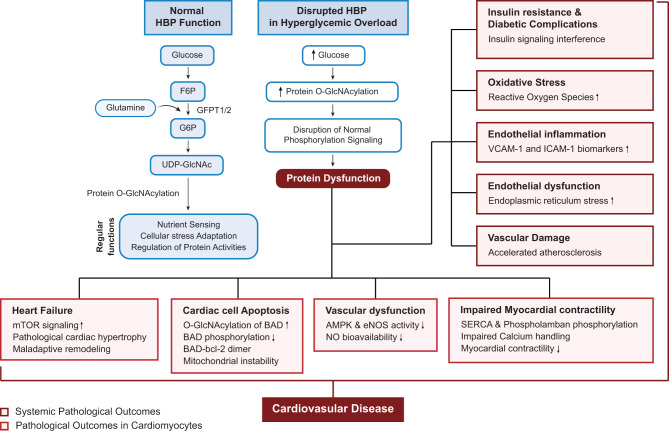
Disruption of the hexosamine biosynthetic pathway and its contribution to cardiovascular disease in hyperglycemia. Under normal conditions, the HBP converts glucose to UDP-GlcNAc via enzymes including GFPT1/2, supporting protein O-GlcNAcylation involved in nutrient sensing, stress adaptation, and protein regulation. In hyperglycemia, excessive glucose flux through the HBP increases O-GlcNAcylation, disrupting phosphorylation signaling and leading to protein dysfunction. This dysregulation contributes to systemic effects such as insulin resistance, oxidative stress, endothelial inflammation, and vascular damage. In cardiomyocytes, it promotes pathological remodeling, mitochondrial instability, apoptosis, impaired calcium handling, and reduced contractility, ultimately driving cardiovascular disease in diabetes. Abbreviations: AMPK, AMP-activated protein kinase; BAD, Bcl-2-associated death promoter; bcl-2, B-cell lymphoma 2; eNOS, endothelial nitric oxide synthase; F6P, fructose-6-phosphate; GFPT1/2, glutamine:fructose-6-phosphate amidotransferase 1/2; HBP, hexosamine biosynthetic pathway; ICAM-1, intercellular adhesion molecule-1; mTOR, mammalian target of rapamycin; NO, nitric oxide; O-GlcNAc, O-linked N-acetylglucosamine; SERCA, sarcoplasmic/endoplasmic reticulum Ca^2+^-ATPase; UDP-GlcNAc, uridine diphosphate N-acetylglucosamine; VCAM-1, vascular cell adhesion molecule-1

The hexosamine biosynthetic pathway (HBP) is an alternative secondary route within glucose metabolism that processes fructose-6-phosphate (F6P) and glutamine into glucosamine-6-phosphate (G6P) through the enzymatic action of glutamine–fructose-6-phosphate transaminase (GFPT), present in the forms of GFPT1 and GFPT2, with GFPT2 being the primary isoform active in cardiac tissue [90]. The activation of HBP ultimately results in the formation of uridine diphosphate N-acetylglucosamine (UDP-GlcNAc), the HBP end product and an essential nucleotide sugar involved in the regulation of highly inducible, dynamic, and reversible O-linked N-acetylglucosamine (O-GlcNAc) protein modifications (Fig. 7) [90]. Under normal circumstances, O-GlcNAcylation functions as a sensor for nutrient availability and cellular stress, modulating the stability, distribution, and activity of many proteins within the nucleus and cytoplasm [91]. This modification responds to increased glucose levels as well as diverse environmental challenges such as ischemia, oxidative damage, low oxygen conditions, changes in osmotic pressure, and exposure to ultraviolet radiations [92]. O-GlcNAc transferase (OGT) serves as the key enzyme that facilitates the binding of O-GlcNAc to serine or threonine residues on proteins, influencing protein function either by directly changing the protein’s activity or indirectly by affecting other regulatory sites through spatial or electrical interference. Conversely, O-GlcNAcase (OGA) counteracts OGT by removing O-GlcNAc groups from proteins. The balance between the activities of OGT and OGA, along with the role of GFAT1/2 enzymes, governs the shift between cardiac protection and the onset of dysfunction (Fig. 8) [93, 94].

In hyperglycemic conditions, excess glucose is diverted into the HBP, resulting in its overactivation beyond normal physiological levels. This heightened HBP activity increases protein O-GlcNAcylation, which disrupts normal phosphorylation and impairs protein function [95]. A key consequence is the development of insulin resistance, oxidative stress, and inflammation-driven vascular complications [96, 97]. HBP overactivation also leads to endothelial dysfunction, endoplasmic reticulum stress, and accelerates atherosclerosis, contributing to cardiovascular damage [97]. In cardiac tissue, it reduces calcium-handling proteins like sarco/endoplasmic reticulum Ca^2 +^ -ATPase (SERCA) and lowers phospholamban phosphorylation, weakening myocardial contractility. Additionally, it promotes apoptosis by enhancing O-GlcNAcylation of BAD, reducing its phosphorylation, and destabilizing the mitochondrial membrane [95]. Extensive evidence highlights the destructive role of prolonged HBP activation and O-GlcNAcylation in cardiac dysfunction. Rajamani and Essop. [97] demonstrated that hyperglycemia triggers extensive activation of the HBP, promoting myocardial cell death. Using H9c2 cardiomyoblasts exposed to high glucose (33 mM), they observed increased ROS, elevated O-GlcNAcylation, and enhanced modification of the pro-apoptotic protein BAD. These molecular changes led to greater BAD-Bcl-2 dimer formation, a known initiator of apoptosis. HBP inhibitors and antioxidant therapies successfully attenuated these harmful effects on cardiac cells [97]. Several clinical observations, including those in idiopathic pulmonary arterial hypertension, also have associated the increased HBP activity and elevated HbA1c levels with impaired cardiac function [98]. Experimental studies further support that excessive HBP flux contributes to cardiac decline, partly through altered O-GlcNAcylation of proteins including AMPK and eNOS [99]. Persistent HBP activation has also been shown to induce pathological cardiac hypertrophy through sustained elevated levels of proteins such as the mechanistic or mammalian target of rapamycin (mTOR), a crucial kinase involved in regulation of cell growth and metabolism signaling. Tran et al. [100] observed that deficiency of GFAT1 provides cardio-protective effects by reducing mTOR activity, while upregulation of HBP under hemodynamic stress contributes to maladaptive cardiac remodeling and heart failure via continuous activation of mTOR pathway.

### Epigenetic modifications

In the early stages of DM without adequate glycemic control, hyperglycemia initiates a cascade of cellular alterations that tend to persist even after subsequent improvements in glycemic control. This persistence reflects the ability of cells to retain the imprint of prior hyperglycemic exposure, thereby sustaining an altered metabolic state that continues to drive long-term cardiovascular risk, a phenomenon termed ‘metabolic memory’ or ‘the legacy effect’ [101]. Achieving glycemic control at an early stage substantially mitigates the likelihood of adverse cardiovascular outcomes, as supported by the clinical studies. Results from the landmark DCCT/EDIC trial revealed that 6.5 years of intensive therapy in T1DM was associated with a 30% sustained reduction in cardiovascular events and a 32% reduction in major cardiovascular events (MACE) over 30 years of follow-up [102]. Similarly, the UKPDS trial demonstrated that early intensive intervention in T2DM led to a 15% reduction in MI and a 13% reduction in all-cause mortality, with benefits that continued over time [103]. A recent meta-analysis including 7 randomized controlled trials and over 40,000 patients demonstrated that intensive glucose-lowering therapy significantly reduced the incidence of MACE, with the greatest benefit observed in individuals with a shorter DM duration ( < 10 years) and no prior CVD [104]. Complementary observational studies further support the legacy effect, demonstrating that higher HbA1c levels within the first year of diagnosis are associated with a substantially increased long-term risk of MACE [104]. These findings suggest that the consequences of hyperglycemia extend beyond immediate biochemical disturbances, potentially involving more fundamental mechanisms such as epigenetic modifications that sustain cellular dysfunction.

Epigenetic modifications are reversible and potentially heritable alterations in gene expression that occur independently of changes in the DNA sequence. These modifications are influenced by both genetic predisposition and environmental exposures, playing a critical role in the regulation of gene activity required for normal development, cellular differentiation, and physiological homeostasis. Dysregulation of these modifications, however, has been strongly implicated in the pathogenesis of metabolic and cardiovascular disorders [105]. The key epigenetic regulatory mechanisms including DNA methylation, post-translational histone modifications, and the functions of non-coding RNAs, are critically involved in the initiation, maintenance, and progression of microvascular and macrovascular complications in DM [106, 107].

#### DNA methylation

DNA methylation, an epigenetic modification involving the addition of a methyl (CH₃) group to the cytosine base of DNA, predominantly at cytosine-guanine (CpG) dinucleotide sites [108], is the most extensively studied epigenetic mechanism, valued for its role in regulating gene expression and the availability of assays that allow large-scale quantification across the epigenome [109]. While the majority of CpG sites across the genome are extensively methylated [110], CpG-rich regulatory regions, particularly CpG islands at gene promoters, generally remain unmethylated to permit active transcription [111]. Aberrant hypermethylation of promoter regions results in gene silencing, either by preventing transcription factor binding or by facilitating the recruitment of repressor complexes, whereas hypomethylation may inappropriately activate normally silenced genes, thereby promoting genomic instability and dysregulated gene expression [110]. DNA methylation may thus either repress or facilitate gene transcription, depending on the specific region and the extent of methylation [112]. The process is catalyzed by DNA methyltransferase (DNMT) enzymes, primarily DNMT1, DNMT3A, and DNMT3B, with their activities disrupted by persistent ROS generation, particularly under hyperglycemic conditions, leading to irregular methylation patterns [113]. Chronic hyperglycemia in T2DM has been reported to induce significant alterations in DNA methylation, subsequently resulting in increased risks of cardiovascular dysfunction [111, 114–116] by promoting oxidative stress, atherosclerosis, endothelial dysfunction and dysregulated lipid metabolism.

Thioredoxin-interacting protein (TXNIP) serves as a key indicator of metabolic disturbances, oxidative stress, and inflammation, and is commonly upregulated in CVD [106]. Gibbs et al. [117] examined DNA methylation in the TXNIP gene among individuals with T2DM in New Zealand. Results revealed a significant association between T2DM and hypomethylation at the TXNIP cg19693031 locus, which also served as a sensitive marker for patients with acute coronary syndromes [117]. Similarly, hypomethylation of the TXNIP cg19693031 locus has been shown to contribute to elevated blood pressure [118], increased triglyceride levels, with or without diabetes, and various other cardiovascular complications [119]. Overexpression of TXNIP has been associated with enhanced oxidative stress, mitochondrial and endothelial dysfunction, reduced glucose uptake, as well as the induction of inflammation, fibrosis, and apoptosis across multiple target cells and tissues implicated in diabetic complications, including retinal cells, renal tissue, peripheral nerves, and vascular cells [120]. Hyperglycemic conditions also induce TNFα expression in cardiomyocytes, which alters DNMT activity and promotes methylation of the SERCA2a gene promoter, leading to reduced SERCA2a expression, calcium overload, and subsequent diastolic heart failure [121]. Monkemann et al. [122] demonstrated that DNA methylation of the p21 (WAF1/CIP1) gene, a downstream effector of p53 signaling, represents an early molecular event in the development of hyperglycemia-induced cardiomyopathy in diabetic rats.

Several other genes have emerged as potential epigenetic biomarkers for diabetic cardiovascular complications. A recent study by Hu et al. [114] demonstrated that hypomethylation of VEGFB, PLGF, PLCB1, and FATP4, key components of the VEGFR signaling pathway, may promote endothelial dysfunction, aberrant angiogenesis, and lipid dysregulation, thereby contributing to vascular complications in DM. Similarly, He et al. [115] have identified 23 differentially methylated regions in 25 genes associated with CVD in patients with T2DM. Notably, four of these genes, ARSG, PNPLA6, NEFL, and CRYGEP, were reported for the first time in this context, which may represent novel epigenetic mechanisms contributing to the development and progression of diabetic cardiovascular complications. ATP-Binding Cassette G1 (ABCG1) is a membrane-associated ATP-dependent transporter that actively mediates the efflux of lipids, such as cholesterol, phospholipids, and oxysterols, and is vital for maintaining cellular and systemic lipid homeostasis [123]. DNA methylation in this gene have been increasingly implicated in the association between T2DM and CVD. For instance, in a cohort of 139 individuals, 82.8% of those with ABCG1 promoter hypermethylation were found to have CHD, whereas only 17.4% of individuals without hypermethylation exhibited the condition. Similarly, DM prevalence was markedly higher in the hypermethylated group (26.9%) than in the non-hypermethylated group (6.5%) [124]. Hypermethylation at ABCG1 promoter loci, including cg06500161 and cg27243685, was significantly elevated in epicardial adipose tissue from CAD patients compared to controls [115]. In contrast, Jin et al. [125] in 2023, demonstrated that hypomethylation of ABCG1 in peripheral blood was associated with an elevated risk of CHD, particularly among females, younger individuals, and patients with HF. These findings collectively indicate that alterations in ABCG1 methylation, regardless of direction, may represent a sensitive epigenetic marker of diabetic vascular disease. A recent review by Kaimala et al. [105] offers an in-depth examination of the role of DNA methylation in T2DM and CVD, to which readers are directed for a more detailed analysis.

#### Post-translational histone modification

Histones are the core proteins around which DNA is wrapped to form nucleosomes, and in humans they are classified into five families, H1, H2A, H2B, H3, and H4. Beyond providing structural organization to chromatin, histones serve as key regulators of gene expression through epigenetic mechanisms. Their N-terminal tails are subject to various post-translational modifications (PTMs), including methylation, acetylation, phosphorylation, ubiquitination, sumoylation, and recently identified lactylation, which collectively modulate chromatin structure and transcriptional activity [107, 126]. These modifications can either activate or repress gene transcription, thereby influencing essential cellular processes such as apoptosis, DNA repair, and inflammatory signaling, with significant implications for disease pathogenesis [107]. Among these, histone acetylation, catalyzed by histone acetyltransferases (HATs) and reversed by histone deacetylases (HDACs) [127], has been particularly associated with hyperglycemia-induced endothelial dysfunction and inflammation [128]. In vascular tissues, hyperglycemia frequently induces aberrant HDAC overactivation, leading to the epigenetic repression of antioxidant defense genes and thereby exacerbating oxidative stress. Inhibition of HDAC3 has been shown to ameliorate endothelial dysfunction in T2DM by activating the NRF2 pathway, which in result reduces ROS production and vascular inflammation [128]. Similarly, genetic deletion or pharmacological inhibition of HDAC6 has been shown to confer cardioprotection against myocardial ischemia-reperfusion injury in experimental diabetes by attenuating TNF-α–mediated mitochondrial damage [129]. Furthermore, global vascular lysine hyperacetylation, driven by the acetyltransferase PCAF, has been linked to elevated ROS levels, impaired VSMC-mediated vasorelaxation, and increased blood pressure in the Goto-Kakizaki rat model of advanced T2DM [130]. Notably, pharmacological inhibition of acetylation with garcinol reduced ROS production in VSMCs exposed to high glucose, which underscores the contributory role of lysine acetylation in T2DM-associated vascular dysfunction [130].

Conversely, histone methylation, mediated by methyltransferases (KMTs and PRMTs) and reversed by demethyltransferases (DMTs), alters chromatin structure to either activate or repress gene transcription depending on the specific amino acid residues modified [113]. For instance, in VSMCs from diabetic db/db mice, miR-125b–mediated suppression of Suv39h1 decreases the repressive histone mark H3K9me3 at inflammatory gene promoters, leading to a persistent pro-inflammatory and pro-atherogenic phenotype that endures even under normoglycemic conditions [131]. In cardiomyocytes treated with high glucose, similar Suv39h1-mediated PTMs were observed at the IL6 promoter, accompanied by a significant reduction in H3K9me3 levels, which persisted even after removal from hyperglycemic conditions [132]. The authors documented histone modifications, rather than mitochondrial dysfunction or apoptosis, as key mediators of metabolic memory in cardiomyocytes exposed to high glucose [132]. Additionally, hyperglycemia has been shown to enhance the repressive histone mark H3K27me3 in human vascular endothelium through upregulation of the methyltransferase EZH2 and suppression of the demethylases UTX/JMJD3, resulting in downregulation of antioxidant genes (e.g., SOD1, SOD2) and elevated NOX4-mediated ROS production [133]. These effects were reversed by pharmacological inhibition of EZH2 with GSK126, which restored endothelial function in both in vitro and in vivo models. In parallel, Xu et al. [134] in 2025 demonstrated that overexpression of BDH1, a key enzyme in ketone metabolism, mitigates diastolic dysfunction, apoptosis, fibrosis, and inflammation in diabetic hearts, whereas BDH1 deficiency exacerbates these pathological features. Mechanistically, BDH1 reduced H3K9 β-hydroxybutyrylation at the LCN2 promoter, suppressed LCN2 transcription and NF-κB activation, while treatment with the β-hydroxybutyrylation inhibitor A485 similarly alleviated the cardiac damage [134].

#### Non-coding RNAs

Non-coding RNAs (ncRNAs) represent a broad category of RNA molecules that lack the protein coding potential but instead regulate gene expression at several levels. Among them, microRNAs (miRNAs) and long non-coding RNAs (lncRNAs) are well-characterized and have been closely associated with the onset and progression of various CVDs [113]. miRNAs are short, ~20–22 nucleotide sequences that interact with the 3′ untranslated regions (UTRs) of target mRNAs, which results in gene silencing through translational repression, mRNA degradation, or sequestration [135], while simultaneously regulating the expression of multiple RNAs [136]. Dysregulated expression of miRNAs has been implicated in the pathophysiological mechanisms that contribute to atherosclerosis and other cardiovascular complications, including endothelial dysfunction, abnormal vascular smooth muscle cell proliferation and migration, altered macrophage activity, and foam cell formation [137]. In endothelial cells exposed to hyperglycemia, NF-κB activation induced persistent up-regulation of miR-27a-3p, which suppressed NRF2, reduced NO production, increased ROS generation and TGF-β signaling, and triggered endothelial-to-mesenchymal transition (EndMT), driving perivascular fibrosis and cardiac dysfunction [138]. The effects were reversed by miR-27a-3p inhibition and NRF2 activator. In human aortic endothelial cells, exposure to high glucose followed by normoglycemic exposure resulted in persistent upregulation of miR-125b and miR-146a-5p, accompanied by sustained NF-κB activation and endothelial dysfunction [120]. Conversely, miR-34a and miR-125b were found to be downregulated in human diabetic hearts, whereas high-glucose exposure in rat cardiomyocytes increased their expression along with glucose metabolism. Overexpression of these miRNAs protected cardiomyocytes from hyperglycemia-induced cell death through direct targeting of HK2 and LDHA, thereby mitigating diabetic cardiomyopathy [139]. Hyperglycemia-induced miRNA dysregulation has also been implicated in ectopic vascular calcification, a hallmark of diabetic vascular disease. In individuals with T2DM, coronary calcification correlated with elevated RUNX2 expression and reduced SIRT7, while mechanistic studies revealed that high glucose induced miR-125b-5p via the JAK/STAT pathway, which suppressed SIRT7 and promoted calcification by myeloid cells [140]. In addition to upregulating several pathogenic miRNAs, hyperglycemia also induces the downregulation of certain cardioprotective miRNAs such as miR-126, miR-130a, miR-134, miR-145, miR-26a, miR-223, and miR-146a, thereby exacerbating various cardiovascular complications including endothelial dysfunction, vascular remodeling, thrombosis and atherosclerosis [136]. Moreover, enhanced glucose flux through the polyol, PKC, and hexosamine pathways, along with AGE formation, drives excessive ROS generation, as previously described. Interactions between cardiac miRNAs and ROS have been demonstrated across various cardiovascular events, including DbCM, atherosclerosis and MI in animal models, where they drive necrosis, fibrosis, apoptosis, hypertrophy, and proliferation, in VSMCs, cardiomyocytes, endothelial cells, and cardiac fibroblasts [136].

In contrast, lncRNAs, previously regarded only as transcriptional byproducts, are now established as important epigenetic regulators of gene expression, and growing evidence implicates their dysregulation in the development of diabetic cardiovascular complications [141]. lncRNAs are typically longer than ~200 nucleotides, regulate gene expression and influence cellular functions through diverse mechanisms, including modulation of chromatin architecture via cis- and trans-interactions with chromatin-associated proteins, engagement with RNA-binding proteins and enhancers, and interference with microRNA activity [142]. DbCM is associated with significant downregulation of the mitochondrial lncRNA MALAT1 in both human and mouse hearts, leading to impaired mitochondrial function and cardiac dysfunction. MALAT1 interacts with miR-320a to regulate mitochondrial gene MT-ND1, and its inhibition enhances miR-320a activity, disrupts Complex I function, and exacerbates systolic and diastolic deficits, whereas MALAT1 overexpression mitigates these effects [143]. lncRNAs also contribute to the recruitment of leukocytes and the formation of macrophage-derived foam cells in DM. For instance, lnRNAs RP5-833A20.1 and E330013P60 are upregulated in foam cells and macrophages under hyperglycemic conditions, which increases the expression of inflammatory cytokines, including IL-1β, IL-6, and TNF, and promotes pro-inflammatory macrophage phenotypes through mechanisms involving miR-382-5p induction and NFIA suppression [141]. In individuals with T2DM and lower extremity arterial disease (LEAD), lncRNA MALAT1 and NLRP3 expression were significantly elevated compared to controls, with MALAT1 levels showing a positive correlation with NLRP3 [144]. Their combined predictive accuracy for LEAD (AUC ≈0.898) identifies MALAT1 as a potential biomarker for diabetic vascular disease. In a rat model of DbCM, lnRNA H19 expression was markedly reduced, whereas H19 overexpression attenuated autophagy and improved ventricular function. Mechanistically, H19 bound to EZH2, enhanced H3K27me3 occupancy at the DIRAS3 promoter, suppressed DIRAS3 expression, and promoted mTOR activation [145]. While in diabetic mice and high-glucose–treated cardiac fibroblasts, expression of the lnRNA *Airn* was markedly reduced, and its overexpression via AAV9 restored cardiac function, prevented fibrosis and myofibroblast activation by stabilizing p53 mRNA in an m6A-IMP2 dependent manner [146]. Together, aberrant DNA methylation, dysregulated histone post-translational modifications, and altered non-coding RNA expression converge to disrupt normal cardiovascular physiology as well as the cardiac transcriptional landscape, driving maladaptive gene expression that promotes fibrosis, inflammation, and apoptosis in diabetic heart disease.

## Potential therapeutic interventions

### Polyol pathway inhibition

Targeting the polyol pathway has emerged as a promising therapeutic strategy for attenuating diabetic cardiovascular complications. Recent research highlights that inhibition of its rate-limiting enzyme, AR, may substantially mitigate vascular dysfunction and related complications in DM [19, 147]. A range of AR inhibitors have been developed to date, including zopolrestat, tolrestat, ranirestat, and caficrestat (AT-001), a highly selective, next-generation AR inhibitor, several of which have progressed through preclinical studies and clinical trials. For instance, zopolrestat (1 μM) attenuated ischemia-reperfusion injury in isolated diabetic rat hearts by reducing sorbitol and fructose accumulation, preserving ATP, and restoring redox balance [148], while results from a separate study revealed that it also normalized lactate and glutamate production, corrected the elevated NADH/NAD^+^ ratio, and shifted substrate utilization from fatty acid oxidation (FAO) toward glycolysis [149]. Although, zopolrestat and tolrestat were discontinued due to severe hepatotoxicity and an unfavorable risk-benefit profile observed in phase 2 and phase 3 clinical trials, respectively [150–152], and ranirestat, which also progressed through phase 3 trial, focused primarily on diabetic polyneuropathy rather than cardiovascular outcomes [153]. AT-001 (caficrestat) remains the only novel AR inhibitor currently under rigorous investigation for diabetic CVD and is being developed for diabetic cardiomyopathy (DbCM), a progressive cardiac fibrosis with no available treatments [152].

In vivo, AT-001 treatment in mice with experimental T2DM improved diastolic function and cardiac efficiency by reducing myocardial fatty acid oxidation without affecting glucose oxidation, while also attenuating cardiac fibrosis and hypertrophy [147]. A recent Phase 1/2 clinical trial evaluating AT-001 in patients with T2DM reported significant reductions in sorbitol levels and decreases in NT-proBNP, a well-known biomarker of cardiac stress [152]. The ARISE-HF Phase 3 trial also evaluated AT-001 in patients with DbCM, particularly at high risk of progression to HF [154]. Results revealed that AT-001, at 1500 mg BID, stabilized cardiac functional capacity over 15 months (mean change of −0.01 ml/kg/min), while the placebo group declined (mean change −0.31 ml/kg/min), with a statistically significant benefit in patients not receiving sodium-glucose cotransporter-2 (SGLT2) or Glucagon-like peptide-1 (GLP-1) therapies (0.62 ml/kg/min; *p* = 0.040). Additionally, in this pre-specified subgroup, clinically significant worsening of cardiac functional capacity (≥6%) was higher in the placebo group (46%) than in the AT-001 group (32.7%) (OR: 0.56; 95% CI:0.33–0.96; *p* = 0.04) [154]. The treatment was generally safe and well tolerated. Although the primary endpoint, the LS mean change difference between placebo and high-dose AT-001, was 0.30 and did not reach statistical significance (*p* = 0.19), a prespecified subgroup analysis showed a significant improvement in peak VO₂ for high-dose AT-001 versus placebo, indicating potential benefit in selected patients and supporting the need for further detailed research and clinical trials.

### Advanced glycation end-products reduction

The detrimental role of AGEs in diabetic cardiovascular complications has directed pharmacological research toward mitigating their effects. Over the past few decades, numerous studies have focused on identifying pharmacological agents that can inhibit glycation reactions and their consequences. The major classes examined include AGE formation inhibitors, AGE crosslink breakers, and AGE-RAGE signaling blockers/RAGE antagonists, with particular emphasis on their therapeutic potential in diabetic macrovascular dysfunction [40]. Recent preclinical and clinical data support the promise of several agents. Aminoguanidine was the first identified compound as an endogenous AGE formation inhibitor that acts by trapping α-dicarbonyl intermediates of early glycation, thereby blocking their subsequent reactions with proteins [155]. In a rat model of STZ- induced T2DM, Aminoguanidine significantly reduced cardiac fibrosis, lowered expression of α-smooth muscle actin, NOX4, and Nos2, and attenuated collagen type I deposition via suppression of ERK1/2 and SMAD2/3 signaling through the AGE/RAGE pathway [156]. Whereas prolonged aminoguanidine treatment in STZ-induced diabetic rats significantly attenuated left ventricular hypertrophy (LVH) and provided notable protection against diabetes-related vascular dysfunction, primarily by limiting AGE accumulation on arterial wall collagen [157]. While aminoguanidine has not been evaluated in diabetic CVD in clinical studies, a phase III randomized trial by Bolton et al. [158] demonstrated that AGE inhibition could attenuate major diabetic complications such as nephropathy and retinopathy, though its safety profile limit clinical use.

Compounds structurally related to aminoguanidine, including ALT-946 and OPB-9195, have also been shown to significantly lower serum AGE levels and tissue accumulation, while also reducing albumin excretion, slowing nephropathy progression, and improving blood pressure in animal models [40]. Whereas TM-2002 and LR-90, through their metal-chelating properties, interact with dicarbonyl compounds, thereby functioning as AGE inhibitors and demonstrating beneficial effects on renal and cardiac outcomes in different animal models [40]. Pyridoxamine, a derivative of vitamin B6, is another compound with AGE-lowering properties and has been demonstrated in multiple studies to reduce their accumulation. For instance, in male Sprague-Dawley rats, treatment with pyridoxamine improved survival after left anterior descending coronary artery (LAD) occlusion, lowered plasma AGE levels, reduced left ventricular end-diastolic pressure, and preserved diastolic function by limiting collagen accumulation, particularly cross-linked type I collagen in the peri-infarct region [159]. Similarly, in a Wistar rat model injected with methylglyoxal to mimic AGE accumulation, pyridoxamine treatment decreased glycation, restored survival pathways (Akt, JNK), normalized the Bcl-2/Bax ratio, and reduced apoptosis during ischemia [160]. In parallel, in a recent randomized double-blind, placebo-controlled trial of abdominally obese individuals, pyridoxamine administration over 8 weeks reduced plasma methylglyoxal (MGO), protein-bound AGE MG-H1, and endothelial dysfunction markers such as sVCAM-1 and sICAM-1 [161].

Another therapeutic strategy involves AGE crosslink breakers, which directly cleave established AGE-mediated protein crosslinks, thereby restoring tissue elasticity and reducing vascular stiffness in diabetes-associated cardiovascular complications. Alagebrium (ALT-711), a thiazolium derivative, is the best-known and most widely studied AGE crosslink breaker in clinical research. Preclinical studies, as well as clinical trials, have shown its therapeutic benefits in DM, hypertension, cardiovascular hypertrophy, vascular stiffening, and associated disorders [162]. Similarly, in a STZ-induced diabetic hypertension rat model, ALT-711 combined with nifedipine produced superior outcomes compared to monotherapy by further lowering SBP and DBP, enhancing vasodilatory mediators (prostacyclin, NO), and reducing prepro-endothelin-1 expression [163]. Similarly, in isolated rat carotid arteries. Whereas, in elderly patients with diastolic heart failure, treatment with ALT-711 for 16 weeks demonstrated reduced left ventricular mass and improved diastolic function compared to placebo [164]. TRC4186 is a relatively novel compound with AGE crosslink-breaking potential. In Ob-ZSF1 rats, treatment with the TRC4186 preserved cardiac function by improving diastolic relaxation and systolic emptying, prevented blood pressure rise, lowered BNP and IL-6 expression, and attenuated renal dysfunction, thereby mitigating DbCM and nephropathy [165]. Although AGE crosslink breakers have demonstrated promising therapeutic effects in preclinical models and small-scale clinical studies, large-scale trials specifically evaluating their role in attenuating diabetic cardiovascular dysfunction are still lacking.

As discussed earlier, the AGE-RAGE signaling axis plays a critical role in the pathogenesis of diabetic vascular complications, making it an attractive therapeutic target as well for preventing both the onset and progression of CVDs in T2DM. Recent studies have shown that inhibition of AGE-RAGE interaction attenuates AGE-mediated cardiovascular complications under hyperglycemic conditions. For example, in USP38-TG diabetic mice, pharmacological inhibition of RAGE with FPS-ZM1 attenuated cardiac systolic and diastolic dysfunction by improving LVEF, LVFS, and E/A ratio, reduced myocardial fibrosis, enhanced cardiac electrical stability, and suppressed inflammatory marker expression [166]. Similarly, Sixagliptin, a potent and well-characterized DPP-4 inhibitor, has been shown to exert cardioprotective effects through suppression of the AGE–RAGE signaling pathway. In diabetic STZ-Isoproterenol challenged rats, Saxagliptin primarily attenuated AGE-RAGE signaling and RAGE expression, which was associated with improved cardiac function, reduced oxidative stress, preservation of myocardial structure, and decreased cardiac apoptosis [167]. Whereas Kong et al. [168] recently demonstrated for the first time the ability of polymetformin to block the AGE–RAGE signaling pathway and attenuate AGE-mediated cardiovascular complications. The compound effectively inhibited AGE formation, reduced expressions of RAGE, IL-1β, TNFα, as well as ROS levels, and protected vascular tissues against oxidative damage. Together, these findings indicate that direct AGE inhibition, disruption of AGE cross-linking, and blockade of the AGE–RAGE signaling pathway effectively relieve AGE-mediated cardiovascular complications under hyperglycemic conditions.

### Protein kinase C pathway inhibition

Inhibition of the PKC pathway has emerged as a promising strategy to attenuate diabetic CVDs, with several PKC inhibitors demonstrating efficacy in preclinical and clinical studies. CGP-53353 (DPH-7) is a highly selective inhibitor of the PKC-β2 isoform with cardioprotective effects under hyperglycemic conditions. Wong et al. [169] in 2020 demonstrated that pretreatment with CGP-53353 significantly reduced myocardial infarction, suppressed abnormal autophagy, and restored ischemic postconditioning–mediated cardioprotection in diabetic rats subjected to I/R injury, which indicates that targeting PKC-β2 can attenuate diabetic cardiac vulnerability by modulating autophagy. Similarly, CGP-53353 attenuated hyperglycemia-induced PKCβ2 activation in cardiomyocytes, thereby preserving caveolin-3 expression and restoring Akt/eNOS signaling, which collectively contributed to the protection against cardiac diastolic dysfunction in diabetic conditions [170]. Ruboxistaurin (RBX) is also a well-known selective PKC-β inhibitor with potential therapeutic effects in alleviating cardiovascular complications in diabetic conditions. For instance, in human coronary artery endothelial cells, selective inhibition of PKC-β by RBX during simulated ischemia–reperfusion significantly restored Akt activation, enhanced anti-apoptotic signaling (iNOS, p-BAD), and suppressed pro-apoptotic mediators (p-FOXO, cleaved PARP, and caspase-3), thereby attenuating endothelial injury and supporting cardiovascular protection in both diabetic and non-diabetic settings [171].

In isolated mouse coronary arterioles, acute inhibition of PKC-β with RBX significantly restored endothelium-dependent relaxation and SK-channel–mediated potassium currents after cardioplegic hypoxia–reoxygenation, thereby attenuating microvascular endothelial dysfunction [172]. In STZ-induced diabetic rats, RBX effectively attenuates myocardial hypertrophy and dysfunction by suppressing PKC-β2 activation, reducing NOX-mediated oxidative stress, and normalizing cardiac structure and function, with superior cardioprotection compared to NAC [173]. Furthermore, in a murine model of myocardial ischemia–reperfusion injury, both genetic deletion of PKC-β and pharmacological inhibition with RBX significantly reduced infarct size, improved left ventricular functional recovery, and lowered serum markers of necrosis compared with controls. These effects were accompanied by decreased PKC-β2 translocation, reduced JNK phosphorylation, and diminished caspase-3 activation [174]. A study conducted by Beckman et al. [52] in a randomized, double-blind, placebo-controlled clinical trial demonstrated that acute hyperglycemia significantly impaired endothelium-dependent vasodilation in healthy subjects, whereas treatment with the RBX effectively preserved vascular responses, thereby preventing hyperglycemia-induced endothelial dysfunction. Overall, inhibition of the PKC pathway, particularly through selective blockade of PKC-β isoforms, has shown promise in animal models for alleviating diabetic cardiovascular complications by reducing oxidative stress, enhancing endothelial function, and preserving myocardial integrity; however, clinical evidence specifically confirming these benefits in diabetic CVD remains limited.

### Oxidative stress reduction

Traditional antioxidants mainly neutralize existing ROS and may be inadequate against the persistent ROS generation in the hyperglycemic state of T2DM. Although established antioxidants such as vitamin E, vitamin C, and N-acetylcysteine have shown promise in preclinical studies, they have largely failed to demonstrate significant protective effects in clinical trials. Notably, long-term vitamin E supplementation showed no benefit in MACE in trials like HOPE [83]. Given the need for more precise, cellular, and pathway-specific treatments, various antioxidant therapeutic approaches are being investigated, with several currently in clinical trial phases [175]. Mitochondria-specific interventions, such as regulators of mitochondrial dynamics and mitochondria-targeted antioxidants, as well as NOX inhibitors, XO inhibitors, and NRF2 activators, summarized in Table 1.

**Table 1:** Pharmacological agents targeting oxidative stress in diabetic cardiovascular complications: evidence from preclinical and clinical studies

Class	Study type	Compound	Model	Dose	Duration	Effects observed	Reference
Drp1 inhibitor	in vivo	Mdivi-1	HFD-STZ-induced diabetic mice	1.2 mg/kg	15 mins (prior to reperfusion)	Drp1 translocation ↓ Mitochondrial fission ↓ Mitochondrial/cardiac function ↑ Infarct size ↓ Cardiomyocyte apoptosis ↓ Oxidative stress ↓ Serum troponin I/LDH ↓	[176]
		Mdivi-1/M1	HFD-induced prediabetic Wistar rats	1.2 mg/kg/2 mg/kg	Pre/during ischemia & reperfusion onset	Cardiac mitochondrial ROS↓ Membrane depolarization ↓ Swelling and dynamic imbalance ↓ Arrhythmias/infarct size ↓ LV function ↑	[177]
SGLT2 inhibitors	in vivo	Empagliflozin	HF induced C57BL/6 mice	30 mg/kg/d	8 weeks	Mitochondrial fission ↓ Myocardial fibrosis ↓ Cardiac function ↑	[178]
		Dapagliflozin	Insulin-resistant metabolic syndrome rats	5 mg/kg/d	2 weeks	Restored mitochondrial dynamics protein imbalance Preserved mitochondrial membrane potential Oxidative stress ↓ LV function ↑	[179]
Antioxidants	Cohort	NAC	T2DM patients (*n* = 46,718)	-	13 years	Incidence of MACE ↓ (41.74% vs 46.87%) Adjusted Hazard Ratio for overall MACE ↓ (aHR 0.84, 95% CI 0.81–0.86) Higher cumulative DDD → further MACE risk ↓ (aHR 0.61, 95% CI 0.58–0.64)	[180]
	in vivo	CoQ10	T2DM db/db mice	10 mg/kg/d	10 weeks	Superoxide generation ↓ Diastolic function ↑ Hypertrophy↓ Cardiac fibrosis ↓ Akt phosphorylation ↑	[181]
		MitoQ	HFD-STZ-induced diabetic rats	2.8 mg/kg	15 mins prior to ischemia	CK-MB/LDH ↓ Infarct size ↓ Cardiomyocyte apoptosis ↓ Cardiac function ↑ Mitophagy status ↑ PINK1/Parkin expression ↑	[182]
NOX inhibitor	in vitro	GKT137831	DOX-induced cardiotoxicity in NRCMs	5 µM	1 hour	NRCM viability ↑ ROS ↓ Cardiomyocyte apoptosis ↓ PARP,/CC3/BAX ↓ Bcl-2 ↑ MAPK pathway activation ↓	[183]
	in vivo	GKT137831	DOX-induced cardiotoxicity mice	60 mg/kg/d	6 weeks	LVEF ↑ FS% ↑	[183]
		GKT137831	ApoE−/− mice	60 mg/kg/d	10 weeks	NOX1 ↓ ROS ↓ nitrotyrosine ↓ F4/80 expression ↓ vascular leukocyte adhesion ↓ Proinflammatory and fibrotic markers ↓	[184]
		GKT137831	ApoE−/− mice	30 mg/kg/d	10 weeks	Atherosclerosis development ↓	[185]
XO inhibitors	in vitro	Allopurinol	H9C2	100 µM	48 hours	H9C2 viability ↑ Hyperglycemic hypertrophy ↓ Oxidative stress ↓ Apoptosis ↓ Autophagy ↓	[186]
		Febuxostat	CCs induced Murine macrophages	50/200 µM	10–15 minutes	Uric acid ↓ ROS ↓ IL-1β/MCP-1/IL-1α/IL-6 release ↓	[187]
	in vivo	Allopurinol	STZ-T1DM rats	100 mg/kg/d	4 weeks	Improved cardiac function HR/LVEF/SW ↑ CO/LVVs ↑ Oxidative stress ↓ Apoptosis ↓ Autophagy ↓ NRF2/HO-1↑	[186]
		Allopurinol	BO-mouse	50 mg/kg/d	2 weeks	Cardiac function ↑ XO-mediated ROS ↓ calmodulin kinase II ↓ Normalized phosphorylation of ryanodine receptor 2 and phospholamban	[188]
		Allopurinol	Alloxan-induced diabetic rabbits	60 mg/kg/d	8 weeks	Interstitial fibrosis ↓ Left ventricular hypertrophy ↓ ROS and oxidative stress markers ↓ AF susceptibility ↓ Atrial structure and function ↑ Ca^+2^ handling ↑	[189]
		Febuxostat	ApoE−/− mice	2.5 mg/kg/d	12 weeks	Plasma XO activity ↓ Plaque formation ↓ Arterial ROS levels ↓ Macrophage infiltration ↓ Endothelial function ↑	[187]
	Cohort	Allopurinol/ Febuxostat	High cardiovascular risk patients (*n* = 2,724)	-	2.7 years	CVD incidence ↓ (HR = 0.48, 95% CI 0.26–0.91; p = 0.024)	[190]
		Allopurinol /Febuxostat	Hyperuricemic patients (*n* = 130,092)	-	2.4–4.1 years	CVD incidence ↓ HF, MI ↓ Ischemic stroke risk ↓ Oxidative stress markers ↓ Lower CVD incidence in Febuxostat group (1000 person-year incidence rate: 16.09 versus 16.32) (hazard ratio [HR] 0.93, 95% confidence interval [CI], 0.87–0.99; *p* = 0.028)	[191]
NRF2 activators	in vitro	RTA 408	H9c2 cardiomyocytes (HG)	62.5/125 nM	48 hours	ROS generation ↓ Restored mitochondrial fusion Mitochondrial membrane potential ↑ Myocardial cell apoptosis, Drp1 ↓ Mfn1, Mfn2, and OPA ↑ NRF2/HO-1 expression ↑ IL-6, TNF-α ↓ NF-κB activation ↓	[192]
	in vivo	Sulforaphane	T1DM (OVE26) Mice	0.5 mg/kg 5 days per week	18 weeks	Diabetic cardiomyopathy ↓ Cardiac function ↑ Hypertrophy ↓ Fibrosis ↓ Inflammation ↓ Oxidative damage ↓	[193]
		Sulforaphane	T2DM-induced cardiomyopathy mice	0.5 mg/kg 5 days per week	3 months	Cardiac function ↑ Cardiac remodeling ↓ Inflammation ↓ oxidative damage ↓ NRF2 expression via the AMPK/AKT/GSK3β pathway ↑	[194]
		Bardoxolone methyl	CHF rodents	5 mg/kg/d	2 weeks	Cardiac function ↑ SV and CO ↑ LVEDP ↓ NRF2 expression ↑ Myocardial inflammation↓ Oxidative stress ↓ Antioxidant enzymes (NQO1, HO-1, Catalase) ↑	[195]
		Bardoxolone methyl	WTD-GS rats	3 mg/kg/d	2 weeks	Endothelium-dependent relaxation ↑ ROS production ↓ NOX2 ↓, SOD2 ↑, catalase ↑) NRF2 target genes (NQO1/GSTP1/HMOX1) ↑ VCAM-1 ↓	[196]
		RTA 408	T2DM-induced cardiomyopathy db/db mice	5/10 mg/kg/d	16 weeks	EF/SV/CO ↑ Fibrosis ↓ Myocardial hypertrophy ↓ Mitochondrial function ↑ NRF2 expression↑ Oxidative stress ↓ Inflammation↓ Apoptosis ↓	[192]
	Systematic review and Meta-analysis	Sulforaphane-yielding Broccoli sprouts	T2DM, MetS, HTN, obese adults, healthy overweight adults (*n* = 579)	Sulforaphane yield; 3–65.47 µmol/g	1–12 weeks	SBP ↓ by average of 10.9 mmHg (95% CI: −17.0, −4.86) DBP ↓ by an average of 6.95 mmHg (95% CI: −10.6, −3.28) improved lipid biomarkers	[197]

#### Improving mitochondrial dynamics

Regulation of mitochondrial dynamics for the treatment of cardiovascular dysfunction in DM, particularly in DbCM has been promising in several studies. In myocardial ischemia–reperfusion–induced diabetic mice, inhibition of Drp1 with Mdivi-1 at 1.2 mg/kg 15 minutes prior to reperfusion significantly suppressed mitochondrial translocation of Drp1, limited mitochondrial fission, and enhanced both mitochondrial and cardiac function, while concurrently reducing infarct size, cardiomyocyte apoptosis, oxidative stress, and serum troponin I and LDH [176]. Similarly, in high-fat diet–induced (HFD induced) pre-diabetic rats subjected to cardiac ischemia–reperfusion injury, administration of the mitochondrial fusion promoter M1 (2 mg/kg), either alone or in combination with the Drp1 inhibitor Mdivi-1 (1.2 mg/kg), significantly reduced mitochondrial dynamics imbalance, ROS generation, swelling, and membrane depolarization, which collectively decreased infarct size and arrhythmias while improving left ventricular function [177]. Whereas restoration of Mfn2 expression markedly attenuated mitochondrial fission and halted the progression of DbCM in the hearts of obese diabetic mice. In vitro, Mfn2 reconstitution enhanced mitochondrial membrane potential, mitigated oxidative stress, and promoted fusion-mediated mitochondrial function in cardiomyocytes exposed to high-glucose/HFD conditions, highlighting the regulation of Mfn2 as a promising therapeutic target in DbCM [69].

Several novel hypoglycemic agents such as GLP-1 agonists and SGLT2 inhibitors have also been demonstrated to alleviate mitochondrial dysfunction by balancing the mitochondrial dynamic proteins, exhibiting their vast range of therapeutic effects in diabetic complications. For instance, in patients with T2DM, treatment with semaglutide, a well-known GLP-1 receptor agonist, markedly enhanced mitochondrial dynamics by restoring Mfn2 expression, restored mitochondrial membrane potential, and decreased ROS generation, while concurrently attenuating leukocyte–endothelial interactions and systemic inflammation [198]. In HFD-induced obese insulin-resistant rats subjected to cardiac ischemia–reperfusion injury, dapagliflozin, an SGLT2 inhibitor, exhibited superior cardioprotective efficacy compared with vildagliptin, a DPP-4 inhibitor, as evidenced by suppression of mitochondrial Drp1 expression, reduced infarct size, and improved left ventricular function [199]. While SGLT2 inhibitors have been extensively evaluated for their protective effects in heart failure through large-scale clinical trials such as the EMPA-REG OUTCOME trial, the CANVAS Program, and the DECLARE-TIMI 58 trial, their precise cardioprotective mechanisms remain a subject of ongoing investigation. Emerging experimental evidence has highlighted their ability to restore mitochondrial dynamics, thereby contributing to improved myocardial function and cardioprotection. For example, empagliflozin improved myocardial fibrosis and cardiac function in heart failure mice by modulating mitochondrial dynamics, particularly through inhibition of mitochondrial fission, enhancing energy metabolism, and increasing ATP production [178]. In diabetic mice, empagliflozin improved myocardial microvascular structure and function by activating AMPK, suppressing Drp1 S616 phosphorylation and increasing Drp1 S637 phosphorylation to inhibit mitochondrial fission, which preserved endothelial barrier integrity, reduced mitochondrial ROS production and endothelial senescence, and enhanced microvessel density and eNOS signaling [200]. Similarly, ipragliflozin restored mitochondrial integrity in HFD–fed mice through normalization of OPA1 and Mfn2 proteins, which resulted in preserved structural integrity and reduced oxidative damage to tubular cells [201]. Dapagliflozin preserved mitochondrial membrane potential, corrected fusion–fission protein imbalance, reduced oxidative stress, and improved cardiomyocyte electrophysiology as well as left ventricular function in insulin-resistant metabolic syndrome rats [179]. These findings indicate that improvement of mitochondrial dynamics mitigates diabetic cardiovascular dysfunction, including cardiomyopathy and heart failure; however, the exact contributions of fission and fusion to disease progression and their therapeutic modulation require further clarification through clinical studies.

#### Regulation of mitochondrial oxidative stress

A combined approach to managing oxidative stress and mitochondrial dysfunction appears to be a promising therapeutic strategy for DM-related CVD. Several antioxidants, including N-acetyl-L-cysteine (NAC) and Coenzyme Q10 (CoQ10), a naturally produced endogenous cofactor essential for mitochondrial ETC, have obtained healthcare attention for the management of oxidative stress in diabetic cardiovascular patients. In a STZ-induced diabetic mouse model, NAC treatment significantly reduced oxidative stress and ferroptosis, leading to smaller infarct sizes during myocardial ischemia–reperfusion injury [202]. Similarly, in a recent large population-based cohort of T2DM patients, long-term use of NAC was associated with a significantly lower risk of MACE, with the effect increasing in magnitude at higher cumulative doses [180]. A study conducted by Gibson et al. [203] demonstrated that NAC, at concentrations ranging from 10 to 100 µM levels, attainable in the bloodstream after oral intake, reduced platelet accumulation and enhanced platelet glutathione levels in patients with T2DM, while CoQ10 has been shown to reduce cardiomyopathy and alleviate diastolic dysfunction and cardiac fibrosis in animal models of T2DM [181]. A recent comprehensive review by Samimi et al. [204] demonstrated that CoQ10 supplementation confers antioxidant and anti-inflammatory benefits in both experimental and clinical settings, improves oxidative stress, lipid profiles, blood pressure, and glycemic control, and thus shows strong potential as a multi-modal adjunct therapy in diabetes and its cardiovascular complications.

MitoQ is a mitochondria-targeted form of CoQ10 modified to accumulate more efficiently within mitochondria, where it protects against oxidative stress and preserves mitochondrial function, making it a promising candidate for treating oxidative stress–related conditions such as cardiovascular, and metabolic diseases [205]. For instance, in a type 2 diabetic myocardial ischemia–reperfusion model, MitoQ treatment markedly reduced myocardial injury and apoptosis while enhancing cardiac function and mitochondrial integrity, with cardioprotection mediated through upregulation of mitophagy via the PINK1/Parkin pathway [182]. Additionally, in a randomized, placebo-controlled crossover trial of older adults with impaired endothelial function, MitoQ supplementation was safe and significantly improved vascular endothelial function, reduced arterial stiffness in those with elevated baseline levels, and lowered plasma oxidized LDL [206]. Whereas in an aged rat model of MI, combined supplementation with alpha-lipoic acid and MitoQ significantly enhanced cardiac function while reducing inflammatory cytokines and apoptotic markers [207]. These results collectively indicate that targeting mitochondrial oxidative stress represents a promising therapeutic approach for mitigating diabetic cardiovascular complications through the preservation of mitochondrial function, attenuation of cellular damage, and reinforcement of cardioprotective mechanisms.

#### NOX inhibitors

NOX inhibitors have emerged as a promising strategy as demonstrated in several in vitro and in vivo studies [208]. Various compounds, such as ebselen, CYR5099, apocynin, and setanaxib (GKT137831), have been identified as NOX inhibitors [175], with GKT137831 demonstrating potential efficacy in preclinical studies. In neonatal rat cardiomyocytes subjected to doxorubicin-induced stress, GKT137831 at 5 µM significantly improved cell viability, reduced ROS production, and decreased apoptosis by lowering pro-apoptotic markers (PARP, CC3, BAX) while increasing anti-apoptotic Bcl-2 expression [183]. Similarly, GKT137831 effectively inhibited oxidative stress triggered by hyperglycemic conditions in human aortic endothelial cells (HAECs) in vitro [184]. GKT137831 has also been utilized in animal models to evaluate the effects of pharmacological NOX inhibition on diabetes-associated macrovascular complications [183–185]. In a doxorubicin-induced cardiotoxicity model, GKT137831 at 60 mg/kg/d for 6 weeks significantly improved cardiac function, as reflected by increased left ventricular ejection fraction (LVEF) and fractional shortening (FS%) [183]. Similarly, pharmacological NOX inhibition with GKT137831 at 60 mg/kg daily for 10 weeks from DM onset significantly reduced atherosclerotic plaque formation and progression in diabetic mice. Treated mice showed decreased ROS, lower nitrotyrosine (an oxidative stress biomarker) levels, reduced vascular leukocyte adhesion, diminished macrophage infiltration (F4/80 expression), and decreased proinflammatory and profibrotic markers [184]. Gray et al. [185] further investigated the therapeutic potential of the GKT137831 in reversing established diabetic complications. The compound demonstrated protective effects in diabetic nephropathy by suppressing proinflammatory and profibrotic pathways. In diabetic atherosclerosis, pharmacological NOX1/4 inhibition provided protection primarily at the lower dose (30 mg/kg/day), though to a lesser extent [185]. GKT137831 has demonstrated potential in preclinical studies to reduce cardiovascular complications, but current clinical trials on this agent lack evaluation of macrovascular outcomes, highlighting the need for longer-term studies in this setting.

#### XO inhibitors

XO inhibitors, including the established allopurinol and febuxostat, approved in 1966 and 2009 respectively, were initially developed and approved as uric acid-lowering agents for the prevention and management of gout [209]. In addition to their primary indications, these agent may offer therapeutic potential for managing DM-associated complications because of their ability to reduce ROS production and consequent alleviation of oxidative stress [83]. In vitro, allopurinol enhanced H9C2 cell viability and mitigated hyperglycemia-induced hypertrophy, oxidative stress, apoptosis, and autophagy, effects that were abolished by NRF2 deactivation [186]. While Febuxostat suppressed cholesterol crystals-induced (CCs induced) ROS formation and inflammatory cytokine release in murine macrophages, demonstrating the ability of XO inhibition to mitigate oxidative stress and inflammation at the cellular level [187]. Similarly, preclinical animal studies have demonstrated that XO inhibitors can reduce oxidative stress and improve cardiovascular outcomes. For instance, in STZ-induced T1DM rats, allopurinol at 100 mg/kg/d for 4 weeks improved cardiac function, including heart rate (HR), LVEF, stroke work (SW), cardiac output (CO), and left ventricular end-systolic volume (LVVs) [186]. The treatment also reduced oxidative stress, apoptosis, and cardiomyocyte autophagy, while increasing NRF2 and HO-1 expression. Moreover, in a bite-opening (BO) mouse model, allopurinol administered at 50 mg/kg/day for 2 weeks ameliorated BO-induced cardiac dysfunction, counteracting increased XO-mediated ROS, suppressing calmodulin kinase II activation, and normalizing phosphorylation of ryanodine receptor 2 and phospholamban [188]. In alloxan-induced diabetic rabbits, allopurinol treatment mitigated oxidative stress-mediated atrial remodeling, including interstitial fibrosis, left ventricular hypertrophy, and calcium handling abnormalities. It reduced oxidative stress markers, improved atrial structure and function, and decreased AF susceptibility [189]. Consistently, Febuxostat at 2.5 mg/kg/day for 12 weeks significantly inhibited plasma XO activity, reduced atherosclerotic plaque formation, and decreased macrophage infiltration in the aortic root, indicating protection against atherosclerosis in ApoE−/− mice fed a high-cholesterol diet [187].

Building on these preclinical findings, clinical cohort studies have evaluated the impact of XO inhibitors on cardiovascular outcomes in patients with elevated cardiovascular risk. In a longitudinal cohort study of 2,724 subjects with cardiovascular risk factors (hypertension, diabetes, dyslipidemia, or chronic kidney disease), XO inhibitors’ (allopurinol and febuxostat) use was associated with a significantly lower risk of cardiovascular events over a median follow-up of 2.7 years. After adjustment for covariates, treatment reduced the risk of cardiovascular events (HR = 0.48, 95% CI 0.26–0.91, *p* = 0.024) [190]. Likewise, in a large Taiwanese nationwide cohort study, febuxostat and allopurinol were compared for cardiovascular outcomes in 130,092 hyperuricemic patients over a mean follow-up of 2.4–4.1 years. Febuxostat use was associated with a slightly lower risk of major adverse cardiovascular events, including nonfatal MI, nonfatal ischemic stroke, and cardiovascular death, compared to allopurinol. Subgroup analyses indicated greater benefit in elderly patients, females, and those with DM or CKD, highlighting the potential of XO inhibitors to mitigate oxidative stress-related CVD in patients with hyperuricemia and comorbid DM [191]. However, additional long-term clinical studies are warranted to further substantiate the role of XO inhibitors in the management of DM-associated CVD.

#### NRF2 activators

NRF2 and its suppressor, Kelch-like ECH-associated protein 1 (Keap1), serve as key regulators of cellular redox homeostasis, and have been the focus of extensive recent research [210]. Under physiological conditions, NRF2 is persistently expressed within the cytoplasm, with its function restrained by direct association with Keap1 [211]. Exposure to triggers such as free radicals induces the translocation of NRF2 into the nucleus, where it promotes the upregulation of antioxidant proteins and plays a defensive role against oxidative damage [210]. In T2DM, NRF2 expression is frequently downregulated, resulting in an impaired antioxidant defense and aggravation of oxidative stress. Conversely, activation of NRF2 has been demonstrated to enhance insulin sensitivity, promote insulin secretion, attenuate vascular inflammation, and confer protection against diabetic complications such as CVD, albuminuria, and neuropathy [22]. The activation of NRF2 and subsequent upregulation of antioxidant proteins represent a promising novel therapeutic target for the prevention or management of diabetic complications, particularly where hyperglycemia-induced oxidative stress is closely associated with elevated free radical production. Recent research has demonstrated that multiple bioactive compounds activate NRF2 by interacting with the cysteine residues of Keap1, including sulforaphane, aspalathin, pterostilbene, and bardoxolone methyl [208]. Sulforaphane is a sulfur-rich compound and a major active ingredient of cruciferous vegetables like broccoli. It is also recognized as a potent inducer of endogenous detoxifying enzymes that enhance the body’s defense against ROS and oxidative stress and has been reported to protect against DM-induced cardiovascular dysfunction via activation of the NRF2 pathway [212, 213]. In a type 1 diabetic mouse model (OVE26), administration of sulforaphane at 0.5 mg/kg 5 days per week and zinc at 5 mg/kg for 18 weeks, alone or in combination, significantly attenuated DbCM, as evidenced by improvements in cardiac function, hypertrophy, fibrosis, inflammation, and oxidative damage [193]. Similarly, in a murine model of T2DM-induced cardiomyopathy, sulforaphane at 0.5 mg/kg administered 5 days per week over 3–4 months prevented cardiac dysfunction, remodeling, inflammation, and oxidative damage. Mechanistic analysis revealed that sulforaphane’s cardioprotective effects were mediated through AMPK-dependent improvement of lipid metabolism and activation of NRF2 via the AMPK/AKT/GSK3β pathway [194]. Furthermore, a recent meta-analysis of 10 clinical trials found that dietary supplementation with sulforaphane-yielding broccoli sprouts significantly reduced systolic blood pressure (SBP) by an average of 10.9 mmHg (95% CI: −17.0, −4.86) and diastolic blood pressure (DBP) by 6.95 mmHg (95% CI: −10.6, −3.28), with marginally significant improvements observed in lipid biomarkers [197].

Bardoxolone methyl, a synthetic oleanane triterpenoid initially synthesized for oncological and anti-inflammatory purposes, has subsequently been recognized as a potent pharmacological activator of the NRF2 signaling pathway [214]. Its therapeutic application was further explored in clinical trials involving patients with T2DM and concurrent CKD [211]. Notably, preclinical studies have also demonstrated its efficacy in mitigating cardiovascular disease in diabetic populations. For instance, Tian et al. [195] investigated the therapeutic impact of bardoxolone methyl in a rodent model of chronic heart failure (CHF) induced by MI. Administering 5 mg/kg/d intraperitoneally for 2 weeks, they observed significant improvements in cardiac function, including increased stroke volume (SV) and CO, and reduced left ventricular end-diastolic pressure (LVEDP), alongside elevated NRF2 expression and decreased oxidative stress in non-infarcted myocardium. Mechanistically, bardoxolone methyl enhanced NRF2’s association with CREB-binding protein, upregulated antioxidant enzymes (e.g., NQO1, HO-1, Catalase), and attenuated myocardial inflammation [195]. More recent studies have demonstrated that bardoxolone methyl and its structural analogs confer protective effects against cardiomyopathy in experimental models of glucotoxicity and db/db mice, both in vivo and in vitro [192], and ameliorates DM-induced endothelial dysfunction in WTD-GS rats [196]. Clinical evaluations of bardoxolone methyl in T2DM have yielded mixed results. The BEACON trial (ClinicalTrials.gov ID NCT01351675), involving patients with advanced CKD, was terminated prematurely due to an increased incidence of HF and elevated cardiovascular risk, despite no demonstrable cardiovascular benefit. Conversely, the TSUBAKI study (ClinicalTrials.gov ID NCT02316821), conducted in T2DM with stage 3–4 CKD and a lower baseline risk of HF, reported significant improvements in measured glomerular filtration rate (GFR) without major safety concerns, suggesting that bardoxolone methyl may serve as a therapeutic benefit in carefully selected CVD patient populations [215]. Another NRF2 activator, UD-051, represents the most recent advancement in this area, developed in 2025 by UBE Corporation in collaboration with Kumamoto University [216]. It inhibits the NRF2-Keap1 interaction, alleviating oxidative stress and inflammation. In preclinical studies, UD-051 suppressed the progression to renal failure and significantly extended survival in proteinuric Alport syndrome model mice, particularly when combined with a renin-angiotensin system (RAAS) inhibitor. Although primarily tested for renal disease, its NRF2-mediated mechanism suggests potential therapeutic applications for CVDs by reducing oxidative stress and inflammation in cardiac cells [217].

### Hexosamine biosynthetic pathway inhibition

The HBP represents a key metabolic link between hyperglycemia and cardiovascular dysfunction in DM, making it an attractive therapeutic target. Pharmacological inhibition of HBP has shown promise in experimental studies, where inhibition of GFAT-mediated flux reduced oxidative stress, improved endothelial function, and mitigated pro-apoptotic signaling in diabetic cardiovascular models. In a model of isoproterenol-induced cardiac hypertrophy in mice, administration of the GFAT inhibitor 6-diazo-5-oxo-L-norleucine (DON) significantly blunted hypertrophic growth, reduced protein O-GlcNAcylation as well as Akt activation, without altering overall hemodynamics such as HR and BP [218]. Additionally, in human endothelial cells, AMPK activation was found to phosphorylate GFAT1, the rate-limiting enzyme of the HBP, thereby reducindg its activity and lowering O-GlcNAc levels. This regulatory effect alleviated hyperglycemic impairment of angiogenesis, while pharmacological inhibition of GFAT1 by DON enhanced VEGF-mediated sprouting, which indicates AMPK–GFAT1 signaling as a critical mechanism in protecting against hyperglycemia-induced vascular dysfunction [219].

Obilineni et al. [220], in a recent review, emphasized the critical role of GFAT in diabetic complications and proposed that its inhibition, whether via glutamine analogs, small-molecule inhibitors, natural compounds, isoform- or tissue-specific targeting, feedback regulation, or PKA-mediated phosphorylation, holds promise as a therapeutic strategy for the management of diabetic complications [220]. While Ukwenya et al. [221] identified several compounds from *Anacardium occidentale* as potential GFAT1 inhibitors where kaempferol-3-O-β-d-xyloside and myricetin demonstrated the strongest binding affinities and favorable interaction energies with the GFAT1 active site. These flavonoids exert their inhibitory potential by stabilizing key hydrogen bond interactions within the catalytic pocket, suggesting their promise as natural lead molecules for antidiabetic drug development. Similarly, the inhibition of HBP with azaserine effectively reduced oxidative stress and inflammation, even under persistent hyperglycemic conditions. In human endothelial cells and isolated rat aortas, azaserine, a glutamine analog and inhibitor of GFAT, attenuated hyperglycemia-induced oxidative stress, suppressed VCAM-1/ICAM-1 expression, and improved endothelial relaxation. However, its protective effect was attributed mainly to antioxidant activity, suggesting that azaserine prevents endothelial inflammation and dysfunction under hyperglycemia independently of HBP inhibition [222]. Conclusively, HBP inhibition represents a promising therapeutic approach to mitigate hyperglycemia-induced oxidative stress, inflammation, and vascular dysfunction in diabetic cardiovascular complications.

### Epidrugs

Advances in understanding epigenetic mechanisms in cardiovascular complications have facilitated the development of novel pharmacological agents, known as epidrugs, that target epigenetic pathways. In recent years, a growing number of epidrugs have been introduced to target and regulate epigenetic mechanisms. These include DNA methyltransferase inhibitors (DNMTi), histone deacetylase inhibitors (HDACi), bromodomain and extra-terminal domain inhibitors (BETi), histone acetyltransferase (HAT) inhibitors, sirtuin-activating compounds (STACs), histone demethylase inhibitors (HDMi), and histone deacetylase activators (HDACa), as reported by Prandi et al. [136]. Several recent studies have demonstrated the potential of targeting epigenetic modifications across these classes to alleviate hyperglycemia-induced cardiovascular complications. For instance, in diabetic models of myocardial ischemia–reperfusion injury, genetic deletion or pharmacological inhibition of HDAC6 with tubastatin A reduced TNF-α–mediated mitochondrial damage, restored complex I activity, limited infarct size, and improved both immediate and long-term cardiac function [129]. Inhibition of DNMT-1 by 5-aza-2’-deoxycytidine significantly alleviated diabetes-induced myocardial ischemia/reperfusion injury by suppressing NCOA4-mediated ferritinophagy and reducing ferroptosis, thereby protecting cardiomyocytes [223]. Whereas the the selective BET protein inhibitor apabetalone restored endothelial migration and tube formation in high-glucose–treated endothelial cells, suppressed the upregulation of the anti-angiogenic molecule thrombospondin-1 by preventing BRD4/H3K27ac-mediated chromatin activation and THBS1 transcription, preserved VEGFA signaling, and improved vascular perfusion and limb vascularization in diabetic models of endothelial dysfunction and hind limb ischemia [224]. Similarly, in a cohort of 2,425 patients with T2DM and recent acute coronary syndrome, treatment with apabetalone was associated with a significantly lower rate of first hospitalization for heart failure, fewer total heart failure hospitalizations, and reduced combined incidence of cardiovascular death or heart failure hospitalization compared to placebo [225].

The HAT p300 plays a central role in cardiac hypertrophy and fibrosis by acetylating transcription factors such as GATA4 and activating stress-response genes. The HAT inhibitor curcumin, selective for p300/CBP, has been shown to suppress hypertrophy, reduce fibrosis and apoptosis, improve systolic function, and limit infarct size in experimental models through inhibition of p300 activity and modulation of oxidative stress and cardioprotective signaling [226]. In parallel with identifying novel pharmacological epidrugs, Zhang et al. [227], in a recent study, demonstrated that in hyperglycemic cardiomyocytes and diabetic rat hearts, the expression of histone demethylase KDM3A is persistently elevated, accompanied by reduced levels of the repressive histone mark H3K9me2, increased ROS, elevated inflammatory mediators (IL-6, TNF-α), enhanced apoptosis, and sustained myocardial dysfunction even after glucose normalization. Targeted silencing or complete global knockout of KDM3A expression significantly mitigated oxidative stress, suppressed NF-κB/P65 activation, reduced apoptosis and fibrosis, and improved cardiac function, which indicates the therapeutic potential of HDMi in diabetic cardiovascular injury [227]. SRT2104, a potent small-molecule activator of SIRT1, has demonstrated significant protective effects against diabetic cardiovascular complications in preclinical models. In STZ-induced diabetic mice, long-term treatment with SRT2104 markedly restored aortic SIRT1 protein levels, attenuated oxidative stress, inflammation, and endothelial dysfunction, largely through p53 deacetylation and downstream activation of antioxidant signaling pathways [228]. In line with the findings of Chang et al. [228], short-term clinical trials of SRT2104 in patients with T2DM demonstrated modest improvements in arterial stiffness and lipid profiles but failed to yield significant benefits in cardiovascular outcomes. Collectively, current evidence underscores the therapeutic potential of epidrugs in attenuating hyperglycemic cardiovascular complications, though large-scale, long-term clinical trials remain essential to establish their efficacy and safety for established clinical use.

## Concluding remarks and future directions

The rising global prevalence of T2DM has been closely accompanied by a marked increase in CVD, underscoring the complex and deeply rooted relationship between chronic hyperglycemia and cardiovascular complications. Persistent elevations in blood glucose activate several interconnected biochemical and molecular pathways, including the polyol pathway, the formation of AGEs, activation of protein kinase C, the hexosamine biosynthetic pathway, excessive oxidative stress, and epigenetic modifications. While each of these mechanisms contributes uniquely to cardiovascular dysfunction, their cumulative effect is the overproduction of ROS, creating a pro-oxidative environment that drives endothelial dysfunction, inflammation, and ultimately cardiovascular complications. This concept was first consolidated by Michael Brownlee and colleagues, who between 1995 and 2005 proposed an integrated mechanistic model, describing mitochondrial superoxide overproduction as the central driver of hyperglycemia-induced vascular injury [56, 79, 229, 230]. Their landmark framework brought clarity to what had previously been a fragmented understanding of diabetic complications from 1966 to 1990. Since then, numerous experimental and clinical studies have validated the importance of oxidative stress, while also identifying at least 10 additional ROS-generating sites within mammalian mitochondria [83], thereby expanding the original hypothesis.

Nevertheless, important challenges persist. Although ROS-centered hypotheses have been highly influential, large clinical trials with broad-spectrum antioxidants have produced inconsistent and often disappointing results. For example, the HOPE trial, which evaluated vitamin E as an established antioxidant, failed to demonstrate the anticipated benefits. These outcomes suggest that ROS cannot be effectively targeted by antioxidants alone and that hyperglycemia-induced damage involves far more intricate mechanisms. This recognition has stimulated the development of alternative therapeutic strategies. Current investigational approaches include inhibitors of enzymatic ROS sources, such as NADPH oxidase and xanthine oxidase, pharmacological activators of the antioxidant transcription factor NRF2, and mitochondria-targeted interventions, including the mitochondrial-specific antioxidant MitoQ and modulators of mitochondrial fission and fusion, aimed at mitigating excessive oxidative stress. Additionally, pathway-specific interventions, such as AR inhibitors, AGE cross-linking inhibitors, AGE/RAGE signaling pathway inhibitors, selective PKC-β inhibitors, and HBP inhibitors, have demonstrated potential to suppress oxidative stress and ROS generation from the earliest stages of hyperglycemia-induced vascular damage. In parallel, the concepts of “metabolic memory” and epigenetic regulation have gained considerable attention. Epigenetic mechanisms, including DNA methylation, histone post-translational modifications, and non-coding RNAs, are now recognized as critical drivers of sustained vascular dysfunction, even after glycemic control is achieved. These insights have expanded the scope of current research, indicating that hyperglycemia leaves lasting molecular imprints that conventional therapies cannot fully reverse.

Despite these advances in mechanistic understanding, translating discoveries into effective clinical therapies remains a substantial challenge. At present, only a limited number of established pharmacological classes have demonstrated consistent cardiovascular benefit in patients with T2DM. Among these, SGLT2 inhibitors have shown remarkable efficacy in reducing MACE, including hospitalization for heart failure, atherosclerotic cardiovascular events, sudden cardiac death, and renal complications. Similarly, GLP-1 receptor agonists have exhibited cardioprotective effects, while DPP-4 inhibitors continue to be investigated for their potential supportive roles. Although these agents represent an important step forward, they exert their benefits primarily through systemic effects rather than by directly targeting the fundamental hyperglycemia-driven molecular pathways. Looking ahead, the future of diabetes and cardiovascular research lies in bridging mechanistic insights with therapeutic innovation. Interventions that combine established glucose-lowering strategies with pathway-specific approaches hold considerable promise. For instance, therapies aimed at modifying epigenetic marks or enhancing endogenous antioxidant defenses may offer durable protection against vascular injury. Likewise, precision medicine approaches that integrate genetic, metabolic, and epigenetic profiling could enable earlier identification of high-risk individuals and more targeted therapeutic interventions [231, 232].

In conclusion, more than three decades of research have substantially advanced our understanding of how chronic hyperglycemia accelerates cardiovascular complications in T2DM. From Brownlee’s unifying mitochondrial ROS hypothesis to the recognition of epigenetic regulation and metabolic memory, the field has evolved into a dynamic area of discovery with profound therapeutic implications. Although challenges remain, the continued integration of basic science, translational research, and clinical trials offers hope that future therapies will not only achieve glycemic control but also directly prevent or reverse the vascular injury underlying diabetic cardiovascular disease. The journey is ongoing, but with each decade, progress brings us closer to realizing clinically effective strategies capable of alleviating the devastating cardiovascular burden of T2DM worldwide.

## Data Availability

No datasets were generated or analysed during the current study.

## Abbreviations

DM: Diabetes Mellitus
IDF: International Diabetes Federation
CVD: Cardiovascular diseases
T1DM: Type 1 Diabetes Mellitus
T2DM: Type 2 Diabetes Mellitus
GBD: Global Burden of Disease
ASCVD: Atherosclerotic Cardiovascular Disease
HFPG: High Fasting Plasma Glucose
LDL: Low-Density Lipoprotein
HbA1c: Glycated Hemoglobin
FPG: Fasting Plasma Glucose
EASD: European Association for the Study of Diabetes
ADA: American Diabetes Association
AACE: American Association of Clinical Endocrinologists
CHD: Coronary Heart Disease
PPG: Postprandial Plasma Glucose
NIDDM: Non-Insulin-Dependent Diabetes Mellitus
MI: Myocardial Infarction
OGTT: Oral Glucose Tolerance Test
PKC: Protein Kinase C
HBP: Hexosamine Biosynthetic Pathway
AR: Aldose Reductase
SDH: Sorbitol Dehydrogenase
NADPH: Nicotinamide Adenine Dinucleotide Phosphate (Reduced Form)
GSH: Glutathione
ROS: Reactive Oxygen Species
NO: Nitric Oxide
NAD^+^: Nicotinamide Adenine Dinucleotide
NADH: Nicotinamide Adenine Dinucleotide (Reduced form)
AGEs: Advanced Glycation End-products
CVS: Cardiovascular System
ECs: Endothelial Cells
AF: Atrial Fibrillation
CHF: Congestive Heart Failure
CAD: Coronary Artery Disease
sRAGE: Soluble Receptors for AGEs
esRAGE: Endogenous Secretory RAGE
hsCRP: High-Sensitivity C-Reactive Protein
TNF: Tumor Necrosis Factor
IL-6: Interleukin-6
CML: Nε-(Carboxymethyl)lysine
DAG: Diacylglycerol
PKC: Protein Kinase C
DHAP: Dihydroxyacetone Phosphate
G3P: Glycerol-3-Phosphate
GAPDH: Glyceraldehyde-3-Phosphate Dehydrogenase
GAP: Glyceraldehyde-3-Phosphate
VSMC: Vascular Smooth Muscle Cells
RBX: Ruboxistaurin
ETC: Electron Transport Chain
FADH_2_: Flavin Adenine Dinucleotide (Reduced Form)
ATP: Adenosine Triphosphate
ADP: Adenosine Diphosphate
TGF-β1: Transforming Growth Factor Beta 1
PAI-1: Plasminogen Activator Inhibitor-1
G6PD: Glucose-6-Phosphate Dehydrogenase
PPP: Pentose Phosphate Pathway
NOX: NADPH Oxidase
XO: Xanthine Oxidase
Ang II: Angiotensin II
K_ATP_: ATP-Sensitive Potassium Channel
ONOO^−^: Peroxynitrite
O₂^−^: Superoxide Radicals
eNOS: Endothelial Nitric Oxide Synthase
BH_4_: Tetrahydrobiopterin
NAC: N-acetyl-L-cysteine
CoQ10: Coenzyme Q10
Mito Q: Mitoquinone Mesylate
ALA: Alpha-Lipoic Acid
HBP: Hexosamine Biosynthetic Pathway
GFPT: Glutamine-Fructose-6-Phosphate Transaminase
UDP-GlcNAc: Uridine Diphosphate N-Acetylglucosamine
O-GlcNAc: O-Linked N-Acetylglucosamine
OGT: O-GlcNAc Transferase
OGA: O-GlcNAcase
ER: Endoplasmic Reticulum
SERCA: Sarco/Endoplasmic Reticulum Ca^2 +^ -ATPase
BAD: BCl^-^2-Associated Death Promoter
AMPK: Adenosine Monophosphate-Activated Protein Kinase
mTOR: Mechanistic Target Of Rapamycin
VCAM-1: Vascular Cell Adhesion Molecule-1
ICAM-1: Intercellular Adhesion Molecule-1
